# Models and methods for analysing clustered recurrent hospitalisations in the presence of COVID-19 effects

**DOI:** 10.1093/jrsssc/qlad082

**Published:** 2023-09-06

**Authors:** Xuemei Ding, Kevin He, John D Kalbfleisch

**Affiliations:** Department of Biostatistics, University of Michigan, Ann Arbor, USA; Department of Biostatistics, University of Michigan, Ann Arbor, USA; Department of Biostatistics, University of Michigan, Ann Arbor, USA

**Keywords:** direct standardisation, indirect standardisation, large database, proportional rates, provider profiling, time-varying covariates

## Abstract

Recurrent events such as hospitalisations are outcomes that can be used to monitor dialysis facilities’ quality of care. However, current methods are not adequate to analyse data from many facilities with multiple hospitalisations, especially when adjustments are needed for multiple time scales. It is also controversial whether direct or indirect standardisation should be used in comparing facilities. This study is motivated by the need of the Centers for Medicare and Medicaid Services to evaluate US dialysis facilities using Medicare claims, which involve almost 8,000 facilities and over 500,000 dialysis patients. This scope is challenging for current statistical software’s computational power. We propose a method that has a flexible baseline rate function and is computationally efficient. Additionally, the proposed method shares advantages of both indirect and direct standardisation. The method is evaluated under a range of simulation settings and demonstrates substantially improved computational efficiency over the existing **R** package *survival*. Finally, we illustrate the method with an important application to monitoring dialysis facilities in the U.S., while making time-dependent adjustments for the effects of COVID-19.

## 1 Introduction

In order to identify poor performance and intervene as necessary, indices of a healthcare facility’s performance are computed and compared to a benchmark or norm by the Centers for Medicare and Medicaid Services (CMS).

For facilities with poor outcomes relative to the national norm, surveillance may be reinforced, and financial penalties imposed, in rare cases leading to loss of accreditation. Because these evaluations are high stakes, and the quality assessment depends on the accuracy of the evaluations, it is crucial to use appropriate statistical methods to analyse facility-level outcomes while accounting for differences in the characteristics of treated patients.

This work is motivated by the development of quality measures for dialysis facilities. The Medicare data for 2020 involves 7,979 US facilities and 509,609 patients who were diagnosed with kidney failure prior to 30 September 2020. The quality of dialysis treatment greatly affects dialysis patients’ quality of life.

Hospitalisations are an important indicator of patient morbidity and quality of life. They are also very costly, accounting for approximately 32% of total Medicare expenditures for dialysis patients in the U.S. (United States Renal Data System, 2020). On average, dialysis patients had 1.58 hospital admissions per person-year in 2018 (United States Renal Data System, 2021). The frequency of hospitalisations is thus an important measurement in monitoring dialysis facilities. To accurately assess facility performance and reduce patient hospital admissions, a risk-adjusted standardised measure, the standardised hospitalisation ratio (SHR), has been used by CMS on the dialysis facility reports site (DialysisData, 2022). Numerically, the SHR=O/E, where *O* is the observed number of hospital admissions of a facility and *E* is the expected number of hospitalisations, given patient characteristics, that would be observed if the facility had the same hospitalisation rate as the national norm. An SHR greater than 1.0 indicates that the facility’s hospitalisation rate is worse than expected based on overall national rates with adjustment for the measured characteristics of patients of this facility. In particular, the SHR has been implemented by CMS in the End-Stage Renal Disease Quality Incentive Program (ESRD QIP), a value-based purchasing program that links a dialysis facility’s payment to the quality of the care it provides.

A facility’s ‘expected’ count (the denominator of the SHR), typically stems from a proportional rates model (Kalbfleisch & Prentice, 2002; Lawless & Nadeau, 1995; Lin et al., 2000), which is a recurrent event analogue of the well-known Cox relative risk model (Cox, 1972). While successful for moderate sample sizes and small-dimensional data, existing statistical software for these methods does not scale to our motivating data set because of the large sample size and high-dimensional facility effects. For example, when implementing the proportional rates model, existing software typically requires dividing each patient’s follow-up time into a set of records with at most one recurrent event in each record. Duplicating the covariates in each record is time-consuming and results in a large amount of redundant information. In our motivating example, the data set had 1,007,094 observations and 257 variables, which can become unduly large if further split by events.

Estimation of the effects of many covariates, especially time-dependent ones, and estimation of high-dimensional facility effects further increase the computational burden.

**Figure 1.**
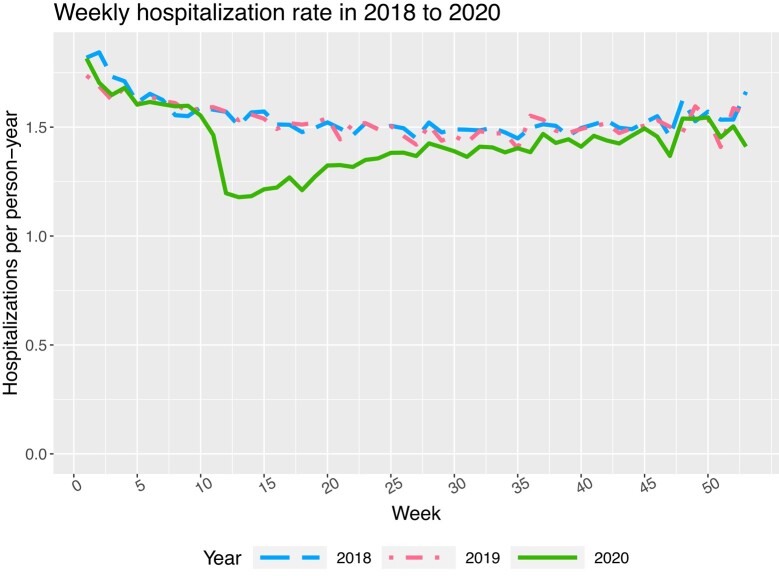
Unadjusted weeklyhospitalisation rates among Medicare dialysis patients at risk on 1 January of each year from 2018 to 2020. The decrease at the end of the year for 2020 is potentially due to reporting delays.

Another important aspect of this example is that patient hospital admissions may vary along two time scales, and modelling these time scales simultaneously adds another level of complexity. First, the frequency of hospitalisation depends on time since the initiation of kidney failure, as shown by Liu et al. (2012), by the United States Renal Data System (2021), and by our estimated model discussed below. Second, the rate of hospital admissions varies with calendar time, with more hospital admissions in winter and fewer in the summer. In addition, in 2020, as an effect of the coronavirus disease 2019 (COVID-19) pandemic, the hospitalisation rate dropped sharply in early spring and gradually picked up in summer. Both the seasonal effect and pandemic impact are evident in Figure 1. Such features require a model to account for the variation over calendar time.

To accommodate high-dimensional clustered recurrent events data, Liu et al. (2012) proposed a two-stage model with a piecewise constant baseline. In Stage I, this method fits a model stratified by facilities, and in Stage II, fits an unstratified model to estimate the overall baseline rate function, using an offset defined with the estimated β obtained from Stage I. This model solves the high-dimensional problem by not estimating facility effects directly but computing the expected number *E* (defined above) using the baseline rate function from Stage II and β from Stage I, and adjusts for the calendar-time effect with some time interval indicators. The two-stage model could allow for a time-varying rate over calendar time by introducing more intervals, but an already very complex data structure would become even more complicated.

The SHR=O/E is an example of indirect standardisation (Breslow & Day, 1985; He & Schaubel, 2014; Inskip, 2005; Roessler et al., 2021), where both *O* and *E* are based on the characteristics of the patients in a facility. Although indirect standardisation is a useful measure for facilities to evaluate themselves or for a governing body to evaluate a facility’s events compared to the national norm, it has been argued that indirect standardised measures for different facilities should not be compared, since each facility-specific estimator is essentially adjusted to a different covariate distribution (Breslow & Day, 1985; He & Schaubel, 2014; Inskip, 2005; Roessler et al., 2021). To overcome this disadvantage, direct standardisation can be used, which gives ${\text{SHR}}^{*}=E^{*}/O^{*}$, where $O^{*}$ is the total observed number of hospitalisations in all facilities and $E^{*}$ is the expected number of hospitalisations in the whole population if all facilities had hospitalisation rates as in the given facility. Because the same standard population is applied to all facilities, direct standardised measures are directly comparable (He & Schaubel, 2014; Inskip, 2005). However, direct standardisation is more difficult for users to understand and may seem less relevant, since the comparison is based on a different patient mix than they treat. In contrast, indirect standardisation is focused on the given patient mix and is more intuitive. Since both ways of standardisation have advantages and drawbacks, it would be useful to have an approach that admits both direct and indirect interpretations.

To address these issues, we propose a computationally efficient modelling procedure for clustered recurrent events data, which considers both the calendar-time effect and time since initiation of kidney failure. It makes the following contributions: First, the proposed method fits in large data settings. It updates the high-dimensional facility effects using a fixed-point algorithm, which works well even when the number of facilities is large, and estimates the model parameters directly without substantial manipulation or replication of the data. This saves computation time and memory. Second, by using calendar time as the baseline, the proposed method captures various trends of hospitalisations in multiple time scales. The baseline rate is a non-parametric function of calendar time that can reflect seasonal or other trends. The time since the start of dialysis is then modelled as a risk adjustment. Third, the proposed model obtains the same facility effect estimates (or SHRs) from indirect and direct standardisation, and thus shares the classic indirect standardisation’s ease of computation and interpretation, but also provides estimates that are comparable across facilities.

The rest of the article is organised as follows: Section 2 presents the model and estimation algorithm. In Section 3, we conduct simulations to evaluate the time-and-memory usage and accuracy of the proposed method. In Section 4, we apply the proposed model and method to a real-data application of hospitalisations of US Medicare dialysis patients in the presence of COVID-19. Some concluding remarks are given in Section 5. Additional simulation results and real-data summary and estimates are relegated to the online supplementary material.

## 2 Model specification and estimation

### 2.1 Set-up and notation

Let j=1,…,F index the facility, with facility size $n_{1},\ldots ,n_{F}$, where *F* is the total number of facilities. Let i=1,…,n index the patient, where $n=\sum_{\;j=1}^{F}n_{j}$ is the total number of patients. Let $B_{i}$, $C_{i}$, and $D_{i}$ denote the calendar time of beginning to be at risk, right censoring, and death for patient *i*, respectively. Patients are said to be ‘left truncated’, if they enter the study after the start of the observation period, which is slightly different from the usual definition. Define the follow-up time $X_{i}=C_{i}\wedge D_{i}$, with a∧b=min(a,b). Define the at-risk process by $Y_{i}(t)=1(B_{i}\leq t\leq X_{i})$, with 1(⋅) being the indicator function and *t* being a time point in a year. Let $N_{i}^{*}(t)$ denote the cumulative number of events for patient *i* in time interval (0,t], and let $N_{i}(t)=\int_{0}^{t}Y_{i}(s)\;dN_{i}^{*}(s)$ denote the counting process for the observed number of events, where $dN_{i}^{*}(t)$ is the number of events of the patient *i* at time *t*. Because the recurrent event of hospitalisation is stopped by the terminal event of death, $N_{i}^{*}(t)=N_{i}^{*}(D_{i}),\;t\geq D_{i}$. Assume one patient can have at most one event at a time; i.e. $dN_{i}^{*}(t)\leq 1$. Let $Z_{i}(t)=(Z_{i1}(t),Z_{i2}(t),\ldots ,Z_{ip}(t){)}^{T}$ be the possibly time-dependent *p*-dimensional covariate vector. Let $G_{i}$ be the facility index associated with patient *i*, and, finally, define $G_{ij}=1(G_{i}=j)$, $N_{ij}(t)=G_{ij}N_{i}(t)$, and $Y_{ij}(t)=G_{ij}Y_{i}(t)$, i=1,…,n,j=1,…,F. Let $N^{j}(t)$ be the counting process for the number of events in facility *j*. Let $dN.(t)=\sum_{\;j=1}^{F}dN^{j}(t)=\sum_{i=1}^{n}dN_{i}(t)$ be the total observed number of events at time *t*.

With reference to Lin et al. (2000), we assume a semi-parametric proportional rates model. Conditional on patient *i* from facility *j* being alive,


(1) $$E\{dN_{i}^{*}(t)|D_{i}\geq t,Z_{i}(t),G_{i}=j\}=\exp\;\left\{\alpha_{j}+Z_{i}^{T}(t)\beta \right\}\;d\mu_{0}(t),$$

where $d\mu_{0}(t)$ is the baseline rate function, $\alpha =(\alpha_{1},\ldots ,\alpha_{F})$ is the facility effect parameter, and $\beta =(\beta_{1},\ldots ,\beta_{p}{)}^{T}$ is the coefficient for covariate effects. Under the assumption of independent left truncation and censoring, we have


(2) $$E\{dN_{i}(t)|Y_{i}(t),Z_{i}(t),G_{i}=j\}=Y_{i}(t)\exp\;\left\{\alpha_{\;j}+Z_{i}^{T}(t)\beta \right\}\;d\mu_{0}(t).$$

### 2.2 Estimation

Fitting risk adjustment models to recurrent event data with very large samples is computationally challenging, especially when the numbers of healthcare facilities and the candidate adjustment covariates are large and time dependent. Most existing statistical methods fail in large-scale settings because of lack of computational power. As there are almost 8,000 facilities in our motivating data set, treating facilities as categorical variables in the model comes with computation problems, and it requires special methods to calculate the inverse of the observed information matrix. The method we propose circumvents including thousands of indicator variables for facilities by using a fixed-point algorithm.

We first estimate covariate effects β with a stratified model, and then update α by an explicit formula in each iteration.

Let $t_{1}<t_{2}<\cdots <t_{M}$ be the unique event time, $t_{1}\geq 0$, and $\tau \geq t_{M}$ be the end of the study period. Let $F_{\;j,m}$ be the set of patients in facility *j* who have an event at $t_{m}$, m=1,2,…,M, and $dN^{j}(t_{m})$ be the number of events in facility *j* at $t_{m}$; i.e. $dN^{j}(t_{m})$ is the cardinality of $F_{\;j,m}$. Let $dN.(t_{m})=\sum_{\;j=1}^{F}\;dN^{j}(t_{m})$ be the total number of events at $t_{m}$.

To estimate β, we account for tied event times using the Breslow (1974) estimator for a stratified model. Any of the standard approximation would work here because the number of events compared to the number of at-risk patients is small.

The estimator is the solution to the following estimating equation:


(3) $$U(\beta )=\sum_{\;j=1}^{F}\sum_{m=1}^{M}\left\{\sum_{i\;:\;i\in F_{\;j,m}}Z_{i}(t_{m})-dN^{j}(t_{m}){\bar{Z}}_{j}(t_{m})\right\},$$

where


$$\begin{array}{rl} & {\bar{Z}}_{j}(t)=S_{j}^{\left(1\right)}(t)/S_{j}^{\left(0\right)}(t),\\ & S_{j}^{\left(r\right)}(t)=\sum_{l=1}^{n}Y_{lj}(t)Z_{l}^{⊗r}(t)\exp\;(Z_{l}^{T}(t)\beta ),\;r=0,1,2, \end{array}$$

$d_{\;j,m}$ $t_{m}$ and for any vector v, $v^{⊗0}=1$, $v^{⊗1}=v$, $v^{⊗2}=vv^{T}$.

Next, given α and β, an estimating equation for $d\mu_{0}(t)$ is $dN.(t)=d\mu_{0}(t)S^{\left(0\right)}(t)$, where $S^{\left(0\right)}(t)=\sum_{i=1}^{n}Y_{i}(t)\exp\;(Z_{i}^{T}(t)\beta +\alpha_{G_{i}})$, which gives rise to the Nelson–Aalen estimate.


(4) $$d{\hat{\mu }}_{0}(t_{m})=\frac{dN.(t_{m})}{S^{\left(0\right)}(t_{m})},\;m=1,\;\ldots ,\;M,$$

and $d{\hat{\mu }}_{0}(t)=0$ elsewhere.

For given $d\mu_{0}(t)$ and β, one can also estimate α from the unbiased estimating equation


(5) $$\sum_{i=1}^{n}\int_{0}^{\tau }dN_{ij}(t)=\exp\;({\hat{\alpha }}_{j})\sum_{i=1}^{n}\int_{0}^{\tau }Y_{ij}(t)\exp\;\left(Z_{i}^{T}(t)\beta \right)d\mu_{0}(t).$$

Plugging in $d{\hat{\mu }}_{0}(t)$ and $\hat{\beta }$, one can obtain


(6) $${\hat{\alpha }}_{j}=\text{log}(O_{j}/E_{j}),$$

where $O_{j}$ is the observed number of hospital admissions of facility *j* and $E_{j}$ is the (estimated) expected number of hospital admissions if the facility *j* has event rates as in the national norm:


(7) $$E_{j}=\sum_{i=1}^{n}\int_{0}^{\tau }Y_{ij}(t)\exp\;\left(Z_{i}^{T}(t)\hat{\beta }\right)d{\hat{\mu }}_{0}(t).$$

However, the baseline rate function $d\mu_{0}(t)$ and facility effect α cannot be identified, because for any solution $d{\hat{\mu }}_{0}(t)$ and $\hat{\alpha }$, $d{\tilde{\mu }}_{0}(t)=xd{\hat{\mu }}_{0}(t)$ and $\tilde{\alpha }=\hat{\alpha }/x$ is also a solution for any positive constant *x*. Therefore, we need an appropriate constraint. The constraint might be, for example, $\sum_{\;j=1}^{F}n_{j}\alpha_{j}=0$, or $\text{median}(\alpha_{j})=0$. We, however, will consider the constraint, $\sum_{\;j=1}^{F}O_{j}=\sum_{\;j=1}^{F}E_{j}$, for which the SHR for the whole population equals to 1. Under this constraint, the baseline rate function $d\mu_{0}(t)$, the limit of $d{\hat{\mu }}_{0}^{\left(s\right)}(t)$ defined below, can be interpreted as the national norm.

Let


$$C^{\left(s\right)}=\frac{\sum_{i=1}^{n}\sum_{m=1}^{M}Y_{i}(t_{m})d{\hat{\mu }}_{0}^{\left(s\right)}(t_{m})\exp\;\{Z_{i}^{T}(t_{m})\hat{\beta }\}}{\sum_{i=1}^{n}N_{i}(\tau )}=\frac{\sum_{k=1}^{F}E_{k}^{\left(s\right)}}{\sum_{k=1}^{F}O_{k}},$$

where the superscript (s) is for estimation from the *s*th iteration. We then estimate ${\hat{\alpha }}_{j}^{\left(s\right)}$ by a fixed-point algorithm


(8) $$\exp\;({\hat{\alpha }}_{j}^{\left(s\right)})=\frac{C^{\left(s\right)}O_{j}}{E_{j}^{\left(s\right)}},$$

which satisfies the constraint $\sum_{\;j=1}^{F}O_{j}=\sum_{\;j=1}^{F}E_{j}^{\left(s\right)}$ in each iteration. In this algorithm, $C^{\left(s\right)}$ converges to 1, and $\exp\;({\hat{\alpha }}_{j})=O_{j}/E_{j}={\text{SHR}}_{j}$. The proposed estimation procedure is summarised in Algorithm 1, where our pre-specified convergence criterion is that the absolute difference in ${\hat{\alpha }}_{j}$ between the current and previous iterations is smaller than or equal to $10^{-6}$, for any *j*.

**Algorithm 1:** The fixed-point algorithm estimation procedure.

1: Estimate β with a stratified model.
2: Initialise at ${\hat{\alpha }}^{\left(0\right)}=0$. For s=0,1,2,…, iterate the following steps:
3: **While** a pre-specified convergence criterion on α has not been reached,
4: Given ${\hat{\alpha }}^{\left(s\right)}$ and $\hat{\beta }$, obtain $d{\hat{\mu }}^{\left(s\right)}(t)$ by equation (4).
5: Given $\hat{\beta }$ and $d{\hat{\mu }}^{\left(s\right)}(t)$, obtain ${\hat{\alpha }}^{\left(s+1\right)}=\text{log}(C^{\left(s\right)}O_{j}/E_{j}^{\left(s\right)})$, where $$C^{\left(s\right)}=\frac{\sum_{i=1}^{n}\sum_{m=1}^{M}Y_{i}(t_{m})d{\hat{\mu }}_{0}^{\left(s\right)}(t_{m})\exp\;\{Z_{i}^{T}(t_{m})\hat{\beta }\}}{\sum_{i=1}^{n}N_{i}(\tau )}=\frac{\sum_{k=1}^{F}E_{k}^{\left(s\right)}}{\sum_{k=1}^{F}O_{k}}.$$ This incorporates the constraint $\sum_{\;j=1}^{F}O_{j}=\sum_{\;j=1}^{F}E_{j}^{\left(s\right)}$.

The log likelihood function of $\alpha_{j}$ is a continuous and concave function, which guarantees the convergence of this fixed-point algorithm (Armijo, 1966; Goldstein, 1967; Wu et al., 2022). We are optimising the log likelihood function, and only use the scaling factor *C* to recenter the estimated facility effects.

### 2.3 Inference

#### 2.3.1 Standard error of $\hat{\beta }$

Since $\hat{\beta }$ is the maximum likelihood estimator (MLE) of the stratified Cox model, which is well studied in the literature, $\hat{\beta }$ is consistent, and the asymptotic distribution is provided by Kalbfleisch and Prentice (2002). Thus, under certain regularity conditions,


$$n^{1/2}(\hat{\beta }-\beta )\;\overset{D}{\to }\;N(0,\Sigma (\beta )),$$

where Σ(β) is the asymptotic variance–covariance matrix.

If we consider a complete intensity model, the asymptotic variance–covariance matrix of $\hat{\beta }$ can be estimated by $I(\hat{\beta }{)}^{-1}$, where $I(\hat{\beta })$ (provided in the online supplementary material) is the observed information matrix for a stratified model evaluated at $\beta =\hat{\beta }$. The standard error of each ${\hat{\beta }}_{k},k=1,\ldots ,p$, is estimated by the square root of the corresponding diagonal entry of $I(\hat{\beta }{)}^{-1}$. This, however, is based on the very strong assumption that given the full history up to time *t*, including the events and the paths of covariates, the rates depend only on the covariates in $Z_{i}(t)$ and therefore often referred to as the naive standard error.

In this analysis, we consider the marginal rate model (Lin et al., 2000). The marginal rate model only models the marginal effect of the covariates but does not condition on the prior events. In this model, we use the sandwich variance estimator of the asymptotic variance–covariance matrix of $\hat{\beta }$


(9) $$\hat{V}=I(\hat{\beta }{)}^{-1}\sum_{i=1}^{n}{\hat{U}}_{i}^{⊗2}I(\hat{\beta }{)}^{-1},$$

where


$${\hat{U}}_{i}=\sum_{m=1}^{M}\{Z_{i}(t_{m})-{\bar{Z}}_{G_{i}}(t_{m})\}\left\{dN_{i}(t_{m})-\frac{dN^{G_{i}}(t_{m})\exp\;\left(Z_{i}^{T}(t)\hat{\beta }\right)}{{\hat{S}}_{G_{i}}^{\left(0\right)}(t_{m})}\right\},$$

${\bar{Z}}_{G_{i}}(t_{m})$ is the weighted average of the covariates of patients in facility $G_{i}$, the facility associated with patient *i*, at $t_{m}$, and ${\hat{S}}_{G_{i}}^{\left(0\right)}(t_{m})$ is a norming factor for facility $G_{i}$ at $t_{m}$, evaluated at $\beta =\hat{\beta }$. The sandwich estimator is a robust version of the estimated variance–covariance matrix for $\hat{\beta }$.

#### 2.3.2 Standard error of $\hat{\alpha }$

From definition (2), we have the following estimating equation:


(10) $$U(\alpha_{j})=\sum_{i=1}^{n}\int_{0}^{\tau }\{dN_{ij}(t)\}-\exp\;(\alpha_{j})\sum_{i=1}^{n}\int_{0}^{\tau }Y_{ij}(t)\exp\;\left(Z_{i}^{T}(t)\beta \right){d\mu }_{0}(t).$$

The negative derivative of equation (10) is


$$I(\alpha_{j})=\exp\;(\alpha_{j})\sum_{i=1}^{n}\int_{0}^{\tau }Y_{ij}(t)\exp\;\left(Z_{i}^{T}(t)\beta \right){d\mu }_{0}(t).$$

Given β and $d\mu_{0}(t)$, as $n_{j}$ increases, $n_{j}^{1/2}({\hat{\alpha }}_{j}-\alpha_{j})$ also converges to a normal distribution with mean 0, and the asymptotic variance of ${\hat{\alpha }}_{j}$ can be estimated by $I(\alpha_{j}{)}^{-1}$.

Since $\exp\;({\hat{\alpha }}_{j})=O_{j}/E_{j}$ and $E_{j}=\sum_{i=1}^{n}\int_{0}^{\tau }Y_{ij}(t)d{\hat{\mu }}_{0}(t)\exp\;(Z_{i}^{T}(t)\hat{\beta })dt$, it follows that $\hat{I}(\alpha_{j})=O_{j}$. Thus, a naive estimate of the standard error of ${\hat{\alpha }}_{j}$ is $1/\sqrt{O_{j}}$.

A robust estimator of the standard error of ${\hat{\alpha }}_{j}$ is the sandwich estimator:


$$V_{j}=I({\hat{\alpha }}_{j}{)}^{-1}\sum_{i\;:\;G_{i}=j}U_{i}({\hat{\alpha }}_{j}{)}^{⊗2}I({\hat{\alpha }}_{j}{)}^{-1}.$$

Denote $\int_{0}^{\tau }\{dN_{ij}(t)\}$ as $O_{ij}$, and $\int_{0}^{\tau }Y_{ij}(t)d{\hat{\mu }}_{0}(t)\exp\;(Z_{i}^{T}(t)\hat{\beta })dt$ as $E_{ij}$. Since $\exp\;({\hat{\alpha }}_{j})=O_{j}/E_{j}$, it follows that


$$V_{j}=\frac{\sum_{i\;:\;G_{i}=j}(O_{ij}-E_{ij}\frac{O_{j}}{E_{j}}{)}^{2}}{O_{j}^{2}}.$$

In this algorithm, we use a constraint so that the proposed method and the standard approach are fitting the same model and optimising the same likelihood function. In our approach, β and $d\mu_{0}(t)$ are estimated from $\sum_{\;j=1}^{F}n_{j}=n$ patients, which converge at the rate of $n^{-1/2}$. In contrast, $\alpha_{j}$ is estimated by a pseudo-MLE (Gong & Samaniego, 1981) based on only $n_{j}$ patients, which converges at the rate of $n_{j}^{-1/2}$; if F is large, then $\hat{\beta }$ and $d{\hat{\mu }}_{0}(t)$ can be considered as fixed and their variation can be ignored, compared to that of ${\hat{\alpha }}_{j}$. We note that, in most profiling applications, the number of facilities and patients is very large compared to $n_{j}$, so that β and $d\mu_{0}(t)$ can be precisely estimated. This assumption is common in provider profiling applications, which usually involve data sets with very large sample sizes (He et al., 2019; Jones & Spiegelhalter, 2011; Kalbfleisch & Wolfe, 2013). If there are only a small number of facilities, the methods can be adapted to account for the variability in the estimates of β and $d\mu_{0}(t)$.

### 2.4 The equivalence between indirect and direct standardisation

As discussed above, it is important to adjust for the differences in patient characteristics before assessing and comparing facilities. An important advantage of the proposed method is that it can be interpreted as either indirect or direct standardisation. This equivalence can be verified as follows:

Let $E_{\left(j\right)}^{*}$ be the expected number of hospitalisations in the whole population if all patients were treated in the given facility *j*. Direct standardisation of facility-specific hospitalisation outcomes is defined as


$$\frac{E_{\left(j\right)}^{*}}{\sum_{k=1}^{F}O_{k}}=\frac{\sum_{i=1}^{n}\sum_{m=1}^{M}Y_{i}(t_{m})d{\hat{\mu }}_{0}(t_{m})\exp\;\{Z_{i}^{T}(t_{m})\hat{\beta }+{\hat{\alpha }}_{j}\}}{\sum_{k=1}^{F}O_{k}}=\exp\;({\hat{\alpha }}_{j})\frac{\sum_{k=1}^{F}E_{k}}{\sum_{k=1}^{F}O_{k}},$$

where we have imposed the constraint $\sum_{k=1}^{F}O_{k}=\sum_{k=1}^{F}E_{k}$. Thus, the direct standardised measure reduces to SHR under the proposed model.

## 3 Simulation study

To compare the performance of the proposed method with the current **R** package *survival* (version 3.2-7), we implemented the proposed algorithm in **R**, with functions written in **Rcpp** language, and assessed its performance using different simulation settings. Because the standard method is not capable of estimating the model parameters in our example data, we carried out simulations on smaller data sets. All scenarios were repeated 1,000 times. While the estimation results from both algorithms were almost the same, the computation time and memory used by our proposed method were much smaller than the *survival* package.

For all the simulations, we had 10 covariates, and the true covariate effects were


$$\beta =(0.1,-0.5,0.3,0.22,0.38,0.1,-0.5,0.3,0.22,0.38{)}^{T}.$$

Five continuous variables were generated from independent normal distributions with mean 0 and variance 0.09, and five binary predictors were generated from independent Bernoulli distributions which take the value 1 with probabilities equal to 0.2, 0.28, 0.36, 0.44, and 0.52. The facility effect $\alpha_{1}=0$ for the first facility, and $\alpha_{j}∼\text{Normal}(0,0.2^{2})$ for the rest. We generated the data to mimic the motivating data example, that patients arrived on various days over the year. The number of days in the study was τ=365. Let $B_{i}$ denote the calendar time of beginning to be at risk. For each *i*, $P(B_{i}=0)=0.8$, and otherwise, $B_{i}$ was chosen randomly from {0,…,τ−1}. Independently of $B_{i}$, The time at risk for the *i*th patient was $t_{i}=\min(\tau -B_{i},t_{i}^{*})$ where $P(t_{i}^{*}=\tau )=0.75$ and otherwise $t_{i}^{*}$ was chosen randomly from {1,…,τ}, so that time to exit was no larger than the number of days in the study. Time to exit $X_{i}=B_{i}+t_{i}$. We assumed entrances happened at the beginning of a day and events and exits happened at the end of a day, with events preceding exits.

For each patient *i* in facility *j*, we generated hospital admissions from a Poisson process with rate $\rho_{i}$ by generating the gap time $T_{i}^{l},\;l=1,2,\ldots ,$ as an exponential distribution with rate $\rho_{i}=\rho_{0}\times \exp\;(\alpha_{j}+Z_{i}\beta )$, with $\rho_{0}=0.003$. This model resulted in an event rate similar to our real data application. $T_{i}^{l},l=1,2,\ldots ,$ was rounded up to the next integer. The process of generating $T_{i}^{l},l=1,2,\ldots ,$ was repeated until $\sum_{l=1}^{L}T_{i}^{l}\geq t_{i}$, for some *L*, and $T_{i}^{L}$ was discarded if $\sum_{l=1}^{L}T_{i}^{l}>t_{i}$.

We consider the following six scenarios:

Scenario 1: Eight different numbers of facilities were tested: 30, 50, 100, 200, 300, 500, 1,000, and 2,000. In each facility, the number of patients followed a Poisson distribution with mean 100.
Scenario 2: The number of facilities was 100, and the number of patients in each facility followed a Poisson distribution with mean from 50 to 5,000.
Scenario 3: With other configurations being the same as in Scenario 2, we fixed the size of the first facility and the mean facility size as 50.
Scenario 4: We explored the accuracy of $\hat{\beta }$ with correlation within patients. With other configurations being the same as in Scenario 3, the events generating model became: gap time $T_{i}∼\exp\;(\rho_{ij})$ for patient *i* in facility *j*, where
  (11) $$\rho_{ij}=W_{i}\rho_{0}\exp\;\left(\alpha_{j}+Z_{i}^{T}\beta \right),$$
  and $W_{i}∼Gamma(1,1)$ was a patient level random effect.
Scenario 5: We fixed the facility effect of the last facility as 0, changing the size of the last facility from 50 to 5,000, as in Scenario 2. Other configurations were the same as in Scenario 3.
Scenario 6: We explored the accuracy of $\hat{\alpha }$ with correlation within patients. The true data generating system was again the equation (11). Other configurations were the same as in Scenario 5.

As an illustration, in Scenario 1, the percentage of patients with at risk time $t_{i}$ smaller than the number of days (365) ranged from 39.74 to 40.06, and the percentage of patients who dropped before the end of the study ($X_{i}<\tau$) ranged from 22.32 to 22.71. A patient on average had 1.25 to 1.38 events, and the average number of events per 365 days was 1.58–1.73. Results in other scenarios were similar and are omitted here. For the simulation and the real data application, we set $\alpha_{j}=-10$ when estimating the effect of facilities with no events, to avoid taking the log of zero.

### 3.1 Evaluation of computation time and memory

We compared the computation time and memory of the proposed method with the *survival* package in Scenarios 1 and 2. Experiments were conducted on an Intel^Ⓡ^ Xeon^Ⓡ^ Gold 6254 quad-processor with max frequency 4 GHz and RAM 576 GB. The computation time and memory used were recorded on **R** (version 3.6.3) using **R** base function *proc.time* and **R** function *profmem* in **R** package *profmem* (version 0.6.0), respectively.

**Figure 2.**
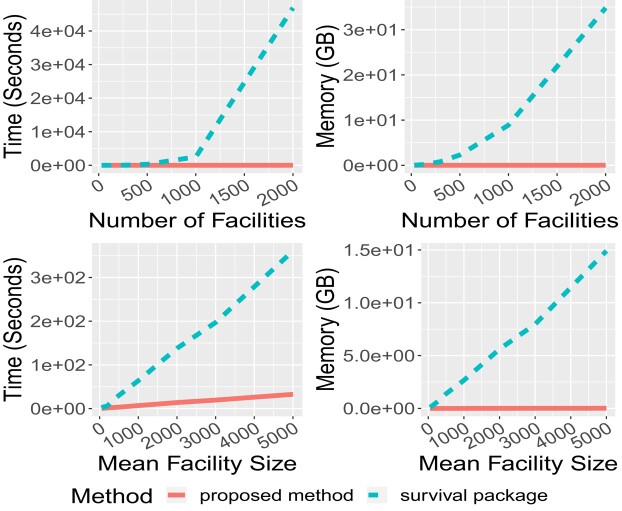
Comparison of the computation time and memory used in the *survival* package and our proposed method. The top two panels are from Scenario 1, in which the mean facility size was fixed at 100, and the number of facilities varied between 30 and 2,000; and the bottom two panels are from Scenario 2, in which the number of facilities was fixed at 100, and the mean facility size varied between 50 and 5,000.

The computation time and memory used are summarised in Figure 2. In both scenarios, our proposed method was substantially faster and required much less memory, compared to the *survival* package; furthermore, as sample size increased, the time used by our method increased more slowly than the *survival* package. When the number of facilities was 2,000, our method was over 3,000 times faster than the *survival* package. In addition, the *survival* package used around 35 GB of memory when the number of facilities was 2,000, which is larger than most computers’ memory. In contrast, we had almost 8,000 facilities in our application, and without revision, the *survival* package could not fit a model on our real data, while our proposed method was able to. Our proposed method was also computationally more efficient when the average facility size increased.

### 3.2 Accuracy of β estimation

We studied the accuracy of estimation of covariate effects in Scenarios 3 and 4. In each repetition, we fitted the model with our proposed method and the *survival* package, and recorded the bias and standard error. A summary of these statistics is listed in Table 1, where the rows of ‘Independent’ are the results of Scenario 3, and the rows of ‘Correlated’ are the results of Scenario 4. Our proposed method had results similar to the *survival* package, with the sandwich asymptotic standard error (sASE) close to the empirical standard deviation (ESD) and a small bias in estimation; however, our proposed method saved computation time and memory and thus could handle a much larger data set. As is typically the case, the robust estimator gave better results when there was correlation. More results can be found in Tables S1 and S2 of the online supplementary material.

**Table 1.** Accuracy of β estimation of the proposed method in Scenario 3 (Independent) and 4 (Correlated)

		Bias	ESD	MSE	ASE	CP	sASE	sCP
Independent	$\beta_{1}$	<0.001	0.043	0.002	0.042	0.941	0.042	0.937
	$\beta_{6}$	− 0.001	0.031	0.001	0.031	0.954	0.030	0.949
	$\beta_{8}$	<0.001	0.026	0.001	0.026	0.948	0.025	0.940
	$\beta_{10}$	− 0.002	0.026	0.001	0.026	0.947	0.026	0.940
Correlated	$\beta_{1}$	− 0.003	0.067	0.005	0.043	0.780	0.069	0.957
	$\beta_{6}$	<0.001	0.053	0.003	0.031	0.743	0.051	0.941
	$\beta_{8}$	− 0.002	0.043	0.002	0.026	0.753	0.042	0.941
	$\beta_{10}$	− 0.003	0.041	0.002	0.026	0.795	0.041	0.952

*Note*. Results were from 1,000 repetitions. ESD = empirical standard deviation; MSE = mean square root error; ASE = the naive estimate of the asymptotic standard error; CP = coverage probability; sASE = sandwich estimator of the asymptotic standard error; sCP = coverage probability based on the sandwich estimator.

### 3.3 Accuracy of α estimation

We were most interested in the accuracy of the estimates of the facility effects. Therefore, in Table 2, we compared the performance of the proposed method and the *survival* package in estimating the effect of the last facility, $\alpha_{F}$, for Scenarios 5 (Independent) and 6 (Correlated). As mentioned above, the facility effects were not completely identifiable since the baseline event rate was flexible. Comparison of the two approaches requires a common choice of reference. In particular, when estimating the facility effects, the ‘factor’ argument in the *survival* package will treat facilities as covariates, and let the first facility have 0 effect (as reference), so we deducted the first facility’s estimated effect from all estimated facility effects. Therefore, both methods satisfied the constraint that the first facility has 0 effect. Once this was done, the two methods gave similar results in the estimation of the facility effects and their standard errors, except that the variance estimates were better with the robust estimates when correlation was present.

**Table 2.** Results of $\alpha_{F}$ estimation using the proposed method in Scenario 5 (Independent) and 6 (Correlated)

	Size	Bias	ESD	ASE	CP	sASE	sCP
Independent	50	0.005	0.212	0.209	0.955	0.205	0.941
	100	0.017	0.195	0.190	0.950	0.185	0.942
	200	0.013	0.186	0.180	0.952	0.176	0.936
	300	0.014	0.179	0.178	0.954	0.174	0.934
	500	0.024	0.176	0.175	0.960	0.171	0.953
	1,000	0.015	0.173	0.172	0.949	0.168	0.932
	3,000	0.011	0.174	0.170	0.944	0.165	0.931
	5,000	0.021	0.175	0.171	0.949	0.166	0.938
Correlated	50	0.011	0.328	0.213	0.805	0.304	0.928
	100	0.019	0.273	0.193	0.841	0.272	0.941
	200	0.002	0.272	0.180	0.820	0.255	0.928
	300	0.018	0.258	0.179	0.828	0.248	0.942
	500	0.032	0.262	0.177	0.812	0.244	0.928
	1,000	0.026	0.257	0.174	0.834	0.237	0.936
	3,000	0.027	0.239	0.172	0.845	0.234	0.945
	5,000	0.030	0.251	0.172	0.824	0.236	0.936

*Note*. Results were from 1,000 repetitions. ESD = empirical standard deviation; ASE = the naive estimate of the asymptotic standard error; CP = coverage probability; sASE = sandwich estimator of the asymptotic standard error; sCP = coverage probability based on the sandwich estimator.

Surprisingly, in Tables 1 and 2, the coverage probabilities of the robust confidence interval tends to be slightly smaller than the nominal level, for which we do not have a reason; nevertheless, as Tables 1 and 2 show, the robust method is much better than the naive approach in the correlated scenario.

## 4 Application

We applied the proposed method to the study of hospital admissions among Medicare patients on chronic dialysis for kidney failure in US Medicare-certified dialysis facilities, derived from the CMS claims and clinical and administrative databases. Our analyses were based on data from the calendar year 2020, with a non-parametric baseline rate function based on the calendar year. This is especially useful for analysing data from 2020, as there was a drop of hospital admissions in early spring of 2020 due to the effect of COVID-19, as elective medical services were delayed, patients and physicians deferred medical care out of caution, and the US government required hospitals to redistribute resources to COVID-19 cases and people to stay at home (Birkmeyer et al., 2020; Kendzerska et al., 2021; The Council of State Governments, 2021). The change over calendar time can be captured by the proposed non-parametric baseline rate function.

The primary outcome of interest was the hospitalisation sequence. Each patient could have multiple hospital admissions (i.e. recurrent events). Hospital admissions were considered to happen at the end of each day, with arrivals and changes of COVID-19 stages preceding any admissions if on the same day. Patients were subject to left truncation if they started to be at risk after the start of the observation period, 1 January 2020. Subjects were followed until the earliest of the following: death, loss to follow-up, 3 days prior to transplant, or 31 December 2020.

The impact of COVID-19 was allowed to vary with time. After exploration, we arrived at this model: We divided the post diagnosis period into four stages and assumed a separate constant effect for each stage; after diagnosis, the patient remained in the ‘COVID’ state until there were no further reported COVID-19 diagnoses for a consecutive 21-day period. We divided this ‘COVID’ state into the first 10 days (COVID1) and the rest (COVID2), with the hypothesis that the impact was most severe during the first few days after infection. After the 21-day period, the patient entered the post-COVID state and remained in that state unless there was another hospitalisation with a COVID-19 diagnosis, in which case he/she became a late-COVID patient. Patients who had an observed previous COVID-19 diagnosis were referred to as in the ‘any-COVID’ group, i.e. the any-COVID group was the combination of the four stages. We assumed that the COVID-19’s interactions with other covariates were the same in all four stages. All patients started in the no-COVID group, i.e. the patients without an observed previous COVID-19 diagnosis. As mentioned above, the Medicare data involve 509,609 patients in 7,979 facilities. Among these patients, 63,521 (12.5%) entered the ‘COVID’ state during the observation period, 34,375 later became post-COVID patients and 1,900 entered the late-COVID state on or before 31 December 2020. More details can be found in Figure S1 of the online supplementary material.

Other variables included in the model were patient age, gender, race (White, Black, Asian/Pacific Islander, and other), ethnicity (Hispanic, non-Hispanic, and unknown), body mass index (BMI) as a categorical variable, time since the onset of dialysis as a categorical variable, proportion of time with Medicare Advantage (MA) coverage, nursing home status, 13 incident comorbidities, 41 conditions for prevalent comorbidities, an indicator of whether a patient had less than 6 months of Medicare coverage in 2019, and some interaction terms and fixed effects for facilities. MA is supplementary insurance to Medicare, and the proportion of time with MA coverage is a time dependent variable that specifies the percentage of time a patient was on Medicare Advantage in the previous 12 months. Nursing home status had three categories: no nursing home care (0 days), short-term nursing home care (1 day to 89 days), and long-term nursing home care (90 days or more). We defined incident comorbidities as those present when the patient first started dialysis and prevalent comorbidities as those observed in inpatient claims within the year 2019. For any patient who had at least six months of claims in 2019, the comorbidities were identified through Medicare claims; for patients with less than 6 months of Medicare coverage in 2019, no prevalent comorbidities were recorded, but an indicator variable accounted for this situation.

While allowing the effect of COVID-19 to be time-dependent, we examined the proportional rate assumption for other covariates graphically by plotting the Schoenfeld residuals for tied data, $s_{k}=\int_{t_{k-1}}^{t_{k}}\sum_{i=1}^{n}[Z_{i}-\bar{Z}(\hat{\beta },s)]dN_{i}(s)$ (Park & Hendry, 2015; Schoenfeld, 1982), over calendar time for each covariate. Taking age as an example, the formula means that for every time point, we sum the age of all patients who have events at that time point, and minus the number of events times the average age of all at-risk patients at that time point. No other important covariates violate greatly the proportional rate assumption. Example Schoenfeld residual plots can be found in the online supplementary material (Figure S2). The variance of the estimated covariate effects was calculated by the sandwich estimator specified in equation (9).

The patients’ characteristics are summarised in Table S5 of the online supplementary material, and the model results are summarised in Table 3 and more completely in Table S6 of the online supplementary material. Table 4 provides a comparison of the main characteristics’ effect in the no-COVID and COVID-19 group, together with the 95% confidence intervals. As expected, the effect of COVID-19 varied significantly in the four stages. A patient in COVID1 had an extremely high relative rate of hospitalisations of 20.18 (p<0.001) compared to patients in the no-COVID group. Patients in COVID2 had a still quite large relative rate of 2.10 (p<0.001). The relative rates for post-COVID and late-COVID patients were respectively 1.37 and 2.04 (p<0.001 for both), still substantial increases in the rate of hospitalisations, as compared to a no-COVID patient.

**Table 3.** Model fitting results for the main covariates

	Hospitalisation			Mortality		
Parameter	Model estimate	*p*-Value	RR and 95% CI	Model estimate	*p*-Value	HR and 95% CI
COVID Stage (reference: no-COVID)	–	–	–	–	–	–
COVID1: first 10 days after COVID diagnosis	3.00	<0.001	${}_{18.83}20.18_{21.63}$	2.47	<0.001	${}_{11.26}11.77_{12.30}$
COVID2	0.74	<0.001	${}_{1.96}2.10_{2.25}$	1.83	<0.001	${}_{5.99}6.25_{6.51}$
Post-COVID	0.31	<0.001	${}_{1.28}1.37_{1.47}$	0.18	<0.001	${}_{1.14}1.20_{1.26}$
Late-COVID	0.71	<0.001	${}_{1.91}2.04_{2.19}$	1.42	<0.001	${}_{3.72}4.14_{4.61}$
Age in 2020 (unit: 20 years, centred at 65, same in age square and the interaction terms)	− 0.05	0.091	${}_{0.89}0.95_{1.02}$	0.58	<0.001	${}_{1.76}1.79_{1.81}$
Age square	0.06	0.035	${}_{0.99}1.07_{1.14}$	0.08	<0.001	${}_{1.07}1.09_{1.10}$
Age * any-COVID	0.07	0.022	${}_{1.00}1.07_{1.15}$	0.00	0.458	${}_{0.97}1.00_{1.03}$
Age square * any-COVID	0.03	0.193	${}_{0.96}1.03_{1.10}$	− 0.01	0.368	${}_{0.96}0.99_{1.03}$
Female	0.07	0.024	${}_{1.00}1.07_{1.15}$	− 0.07	<0.001	${}_{0.92}0.93_{0.95}$
Race (reference: White)	–	–	–	–	–	–
Black	− 0.07	0.022	${}_{0.87}0.93_{1.00}$	− 0.33	<0.001	${}_{0.71}0.72_{0.73}$
Asian/Pacific Islander	− 0.26	<0.001	${}_{0.72}0.77_{0.83}$	− 0.31	<0.001	${}_{0.71}0.73_{0.76}$
Others	− 0.05	0.093	${}_{0.89}0.95_{1.02}$	− 0.20	<0.001	${}_{0.76}0.82_{0.88}$
Ethnicity (reference: Non-Hispanic)	–	–	–	–	–	–
Hispanic	− 0.13	<0.001	${}_{0.82}0.88_{0.95}$	− 0.30	<0.001	${}_{0.72}0.74_{0.76}$
Unknown	− 0.13	<0.001	${}_{0.82}0.88_{0.94}$	− 0.02	0.374	${}_{0.85}0.98_{1.12}$
Cause of ESRD: diabetes	0.02	0.269	${}_{0.95}1.02_{1.10}$	0.11	<0.001	${}_{1.09}1.11_{1.13}$
Asian/Pacific Islander * any-COVID	0.34	<0.001	${}_{1.31}1.40_{1.50}$	0.60	<0.001	${}_{1.67}1.81_{1.97}$
Black * any-COVID	0.09	0.008	${}_{1.02}1.09_{1.17}$	0.16	<0.001	${}_{1.13}1.18_{1.23}$
Race: others * any-COVID	0.11	0.001	${}_{1.04}1.11_{1.19}$	0.29	<0.001	${}_{1.16}1.33_{1.53}$
Hispanic * any-COVID	0.22	<0.001	${}_{1.17}1.25_{1.34}$	0.41	<0.001	${}_{1.44}1.51_{1.59}$
Ethnicity: Unknown * any-COVID	− 0.02	0.305	${}_{0.92}0.98_{1.05}$	− 0.25	0.024	${}_{0.61}0.78_{1.00}$
Female * any-COVID	− 0.11	0.001	${}_{0.83}0.89_{0.96}$	− 0.09	<0.001	${}_{0.88}0.92_{0.95}$
Missing: Primary disease causing ESRD	0.22	<0.001	${}_{1.17}1.25_{1.34}$	0.25	<0.001	${}_{1.11}1.28_{1.47}$
Proportion of days with Medicare Advantages coverage	− 0.08	0.016	${}_{0.87}0.93_{0.99}$	0.07	<0.001	${}_{1.06}1.08_{1.09}$
Time since ESRD (reference: 90 days—1 year)	–	–	–	–	–	–
< 90 days	0.29	<0.001	${}_{1.24}1.33_{1.43}$	0.13	<0.001	${}_{1.11}1.14_{1.18}$
1 year—3 years	0.07	0.027	${}_{1.00}1.07_{1.15}$	0.08	<0.001	${}_{1.06}1.09_{1.11}$
> 3 years	0.11	0.001	${}_{1.04}1.12_{1.20}$	0.35	<0.001	${}_{1.39}1.43_{1.46}$
BMI (reference: ≥ 30)	–	–	–	–	–	–
≤18.4	0.14	<0.001	${}_{1.08}1.15_{1.24}$	0.25	<0.001	${}_{1.24}1.29_{1.34}$
18.5–24.9	0.08	0.008	${}_{1.02}1.09_{1.17}$	0.10	<0.001	${}_{1.09}1.11_{1.13}$
25–29.9	0.05	0.079	${}_{0.98}1.05_{1.13}$	0.04	<0.001	${}_{1.02}1.04_{1.05}$

*Note*. Other coefficients are listed in Table S5 of the online supplementary material.

${}_{a}b_{c}$ means RR (or HR, the hazard ratio) is *b* with (a,c) as the 95% CI. BMI = body mass index; ESRD = end-stage renal disease.

**Table 4.** Estimated relative rate (RR) and 95% confidence interval (CI) for patients with and without a previously observed COVID-19 diagnosis, assuming COVID-19’s interaction effect is the same in all four stages

	No-COVID		COVID-19	
	RR	CI	RR	CI
Age in 2020 (unit: 20 years, centred at 65, same in age square and the interaction terms)	0.95	0.95, 0.96	1.02	1.01, 1.04
Age square	1.07	1.06, 1.07	1.10	1.08, 1.12
Female	1.07	1.06, 1.08	0.95	0.94, 0.98
Race (reference: White)	1.00	–	1.00	–
Black	0.93	0.92, 0.94	1.01	0.99, 1.04
Asian/Pacific Islander	0.77	0.75, 0.79	1.08	1.03, 1.13
Others	0.95	0.92, 0.99	1.06	0.98, 1.15
Ethnicity (reference: Non-Hispanic)	1.00	–	1.00	–
Hispanic	0.88	0.87, 0.89	1.10	1.07, 1.13
Unknown	0.88	0.81, 0.94	0.86	0.74, 1.01

**Figure 3.**
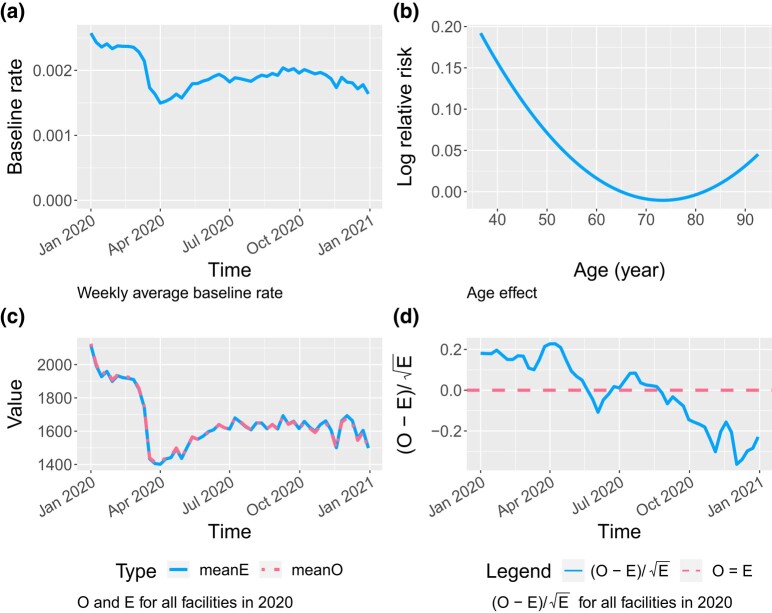
(a) The estimated baseline hospitalisation rate function averaged by week. (b) The estimated age effect curve. (c) Weekly average number of hospital admissions, observed (*O*) and expected (*E*), for all patients. (d) $(O-E)/\sqrt{E}$ plot for all patients. If the model assumption is correct, $(O-E)/\sqrt{E}$ would approximately follow the standard normal distribution.

Among other adjusted variables, age had a U-shaped effect on hospitalisation, where the 65-to-80-year-old patients had the lowest risk. The estimated age effect curve and baseline rate function are provided in Figure 3. Females on average were more likely to be hospitalised (RR = 1.07, p< 0.001 for all comparisons in this paragraph) than males, if they had no COVID-19 history, but their interactions with previous COVID-19 diagnosis had lower risks (RR = 0.89). In the COVID-19 group, the relative rate of females vs. males was smaller than 1 (RR=1.07×0.89=0.95). Similarly, compared to White patients, Black and Asian/Pacific Islander had lower risks of hospitalisations (RR=0.93 and 0.77, respectively) in the no-COVID group, but similar risks (RR=0.93×1.09=1.01 and RR=0.77×1.40=1.08, respectively) in the COVID-19 group; compared to non-Hispanics, Hispanics had a lower relative rate of hospitalisation (RR=0.88) in the no-COVID group, and a relative rate of about 1.10 = 0.88 × 1.25 in the COVID-19 group. Table 4 provides a comparison of the main characteristics’ effect in the no-COVID and COVID-19 group, together with the 95% confidence intervals. While most prevalent comorbidities increased the risks of hospitalisation, the following led to at least a 25% increase in relative rate and had at least 0.5% prevalence in the no-COVID group (RR, prevalence in the no-COVID group): opioid dependence (1.44, 0.7%), pancreatitis (1.44, 0.5%), bipolar disorder (1.30, 0.8%), other liver disease (1.26, 1.0%), diabetes with complications (1.25, 29.4%), and major depressive affective disorder (1.25, 8.1%). Patients with less than 6 months of Medicare coverage in the prior calendar year, 2019, also had a higher risk of hospitalisation (RR: 1.54, 17.4% prevalence in the no-COVID group) due to unknown comorbidities. Patients with 100% Medicare Advantage coverage on average had a lower risk of hospital admissions (RR = 0.93), compared to patients with no Medicare Advantage coverage.

**Figure 4.**
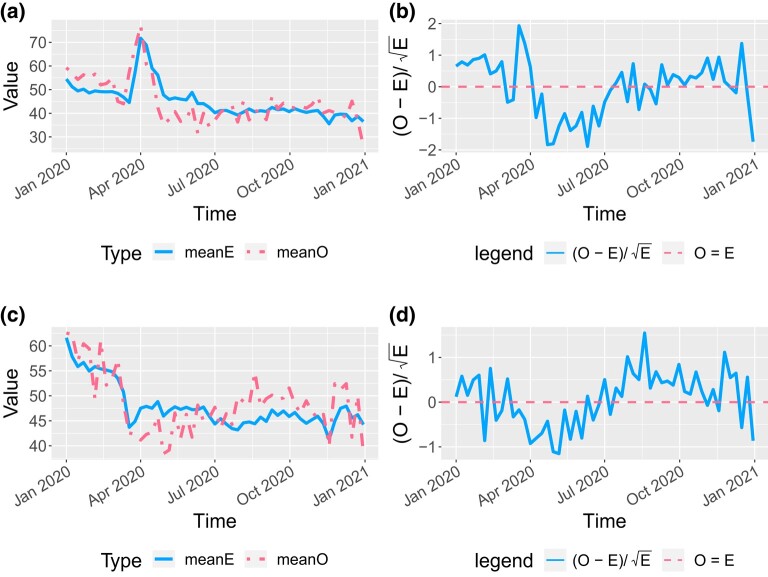
Weekly average number of hospital admissions, observed (*O*) and expected (*E*), for New York City (NYC) and the rest of New York (NY) State. If the model assumption is correct, $(O-E)/\sqrt{E}$ would approximately follow the standard normal distribution. Both NYC and the rest of NY State have this value within ±2, so the variation can be considered due to randomness. (a) *O* and *E* for NYC facilities in 2020, weekly average. (b) $(O-E)/\sqrt{E}$ for NYC facilities in 2020. (c) *O* and *E* for facilities in the rest of NY state in 2020. (d) $(O-E)/\sqrt{E}$ for facilities in the rest of NY.

**Figure 5.**
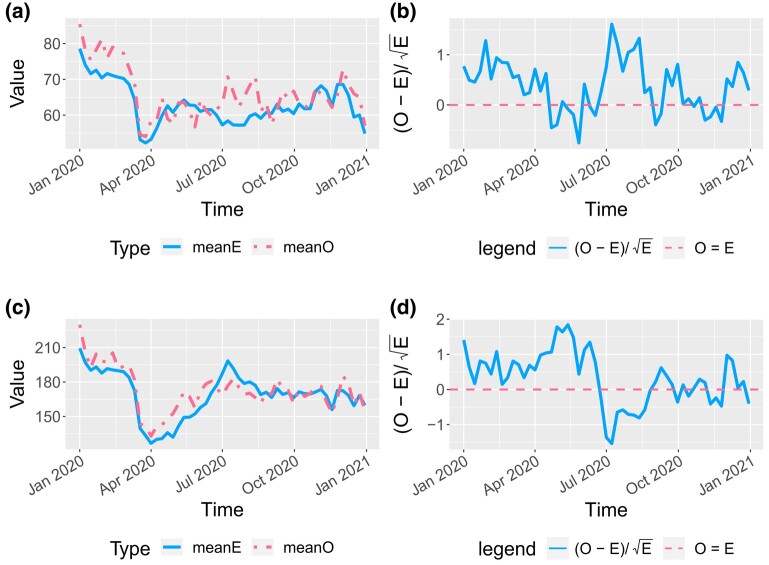
Weekly average number of hospital admissions, observed (*O*) and expected (*E*), for Illinois (IL) and Texas (TX). If the model assumption is correct, $(O-E)/\sqrt{E}$ would approximately follow the standard normal distribution. Both IL and TX have this value within ±2, so the variation can be considered due to randomness. (a) *O* and *E* for IL facilities in 2020, weekly average. (b) $(O-E)/\sqrt{E}$ for IL facilities in 2020. (c) *O* and *E* for TX facilities in 2020, weekly average. (d) $(O-E)/\sqrt{E}$ for TX facilities in 2020.

A weekly averaged *O* and *E* plot over time for all patients is given in Figure 3, where *O* is the observed number of hospital admissions, and *E* is the expected number obtained from the fitted model, assuming that all facilities have the same performance as the national average (i.e. the baseline). A similar *O* and *E* plot is given in Figure 4 for New York City and the rest of New York State. The *O* and *E* matched well for the rest of New York State whereas the prediction for New York City had a short lag. New York City is especially of interest as it was greatly impacted at the beginning of the pandemic and had a sharp increase of hospitalisations in the spring of 2020. Estimation results for other states were also explored. Plots for Illinois and Texas are provided in Figure 5. The fit was very good. If the model assumption is correct, $(O-E)/\sqrt{E}$ would approximately follow the standard normal distribution, so a fluctuation within ±2 can be accepted as due to randomness. The $(O-E)/\sqrt{E}$ in Figures 3–5 is within the random range.

The facility effects are important for surveillance. As an illustration of different facility effects, we provide a histogram and a funnel plot in Figure S3 in the online supplementary material, in which we exclude facilities with expected number of hospitalisations under the null (*E*) greater than 800 or smaller than 5, or the observed number of hospitalisations (*O*) equal to 0.

Computational efficiency is also an important contribution of our analysis. It took less than 17 hours for the proposed method to fit the model. Parallel computing would further shorten the time used, depending on the number of threads used to compute the results. Table S4 in the online supplementary material shows the time, speedup, and efficiency estimated from simulation data, which provides an idea of how fast the algorithm can be when using parallel computation. We also developed code for models without time-dependent covariates; it took about 3 hours to fit the model for the real data.

During the observation period, many patients died, and it is instructive to see what effect COVID-19 had on mortality and to compare the estimates between the hospitalisation and mortality models. A model with the same covariates was fitted with mortality events being the outcome. Most coefficients had a similar pattern as in the hospitalisation model, but there were some differences. The model estimates are shown in Table 3 and in the online supplementary material (Table S6). On one hand, similar to the hospitalisation model, compared to the no-COVID group, COVID1 patients had the highest hazard ratio (11.77, p< 0.001), followed by COVID2 (6.25, p< 0.001), late-COVID (4.14, p< 0.001), and post-COVID (1.20, p<0.001). On the other hand, the baseline hazard of mortality increased in early spring (see Figure S4 of the online supplementary material), while the baseline hospitalisation rate decreased.

## 5 Discussion

In this paper, we proposed a new modelling procedure for clustered recurrent events data. We developed a computationally efficient algorithm for estimation of high-dimensional facility effects and model parameters. The proposed fixed-point algorithm is very fast and provides essentially the same results as the slower partial likelihood approach. The proposed method used calendar time as the baseline time scale, which has only a fixed number of distinct values (365 or 366) and is easier to estimate, and therefore is more suitable for modelling multiple time scales; other time scales, for example, time since the onset of kidney failure, can be readily adjusted for as a categorical covariate. In addition, the proposed model allows a flexible change of baseline rate over time. This analysis also shows the equivalence between the indirect and direct standardisation under a recommended constraint.

The model can be extended to the discrete case (Prentice & Kalbfleisch, 2003). Let $0<t_{1}<t_{2}<\cdots <t_{M}$ be the possible event times and $t_{0}=0$ be the start of the study period. Under the assumption of independent left truncation and censoring, the semi-parametric proportional rates model is


(12) $$P\{N_{i}(t_{k})-N_{i}(t_{k}^{-})=1|Y_{ij}(t_{k}),Z_{i}(t_{k})\}=Y_{ij}(t_{k})\gamma_{0k}\exp\;\left\{\alpha_{j}+Z_{i}^{T}(t_{k})\beta \right\},$$

where $\gamma_{0k}$ is the instant event probability at time $t_{k}$ for a patient with covariates equal to zero and treated by a facility at the national norm. As mentioned above, we assume the number of events for a patient at a time point is at most 1. The discrete model requires some restrictions, usually not of practical importance, in order to assure the probabilities in equation (12) are at most 1. The discrete model (12) would provide results nearly identical to those in the continuous model. Note that the Breslow approximation for ties is exactly correct in the discrete model (Prentice & Kalbfleisch, 2003), and the variance adjustment makes little difference. The equivalence between the indirect and direct standardisation also presents in the discrete model.

We have focused on frequency of hospitalisations, using the number of hospital admissions as our outcome, although the model also works well on analysing other recurrent events, e.g. duration of hospitalisations, transfusion, and emergency department visits.

Our model assumes that the facility effect is constant over the year but in fact, it is possible that the effect varies somewhat; if so, our estimate would be an ‘average’ effect. If this were deemed to be important, it would be possible to allow separate effects for various periods during the year, a possible direction for future work.

Example code is available online at *GitHub* (https://github.com/UM-KevinHe/tdrecur). An **R** package will be uploaded to CRAN.

## Supplementary Material

qlad082_Supplementary_Data

## Data Availability

The data that support the findings of this study were provided by CMS and are not publicly available. Similar data are available through a data use agreement from the United States Renal Data System (USRDS).

## References

[qlad082-B1] Armijo L. (1966). Minimization of functions having Lipschitz continuous first partial derivatives. Pacific Journal of Mathematics, 16(1), 1–3. 10.2140/pjm

[qlad082-B2] Birkmeyer J. D., Barnato A., Birkmeyer N., Bessler R., & Skinner J. (2020). The impact of the COVID-19 pandemic on hospital admissions in the United States. Health Affairs, 39(11), 2010–2017. 10.1377/hlthaff.2020.00980 32970495 PMC7769002

[qlad082-B3] Breslow N. E. (1974). Covariance analysis of censored survival data. Biometrics, 30(1), 89–99. 10.2307/2529620 4813387

[qlad082-B4] Breslow N. E., & Day N. E. (1985). The standardized mortality ratio. In P. K. Sen (Ed.), *Biostatistics: Statistics in biomedical, public health and environmental sciences* (pp. 55–74). North-Holland; Sole distributors for the U.S.A. and Canada, Elsevier Science Pub. Co.

[qlad082-B5] Cox D. R. (1972). Regression models and life tables (with discussion). Journal of the Royal Statistical Society, Series B, 34(2), 187–200. http://www.jstor.org/stable/2985181

[qlad082-B6] DialysisData . (2022). *ESRD Measures*. The University of Michigan Kidney Epidemiology and Cost Center, Ann Arbor. https://www.dialysisdata.org/content/esrd-measures.

[qlad082-B7] Goldstein A. A. (1967). Constructive real analysis. Harper & Row.

[qlad082-B8] Gong G., & Samaniego F. J. (1981). Pseudo maximum likelihood estimation: Theory and applications. The Annals of Statistics, 9(4), 861–869. 10.1214/aos/1176345526

[qlad082-B9] He K., Dahlerus C., Xia L., Li Y., & Kalbfleisch J. D. (2019). The profile inter-unit reliability. Biometrics, 76(2), 654–663. 10.1111/biom.v76.2 31642521 PMC7318309

[qlad082-B10] He K., & Schaubel D. E. (2014). Methods for comparing center-specific survival outcomes using direct standardization. Statistics in Medicine, 33(12), 2048–2061. 10.1002/sim.6089 24436222 PMC4013227

[qlad082-B11] Inskip H. (2005). Standardization methods. In P. Armitage, & T. Colton (Eds.), *Encyclopedia of biostatistics* (Vol. 7, 2nd ed., pp. 5151–5163). Wiley.

[qlad082-B12] Jones H. E., & Spiegelhalter D. J. (2011). The identification of unusual health-care providers from a hierarchical model. The American Statistician, 65(3), 154–163. 10.1198/tast.2011.10190

[qlad082-B13] Kalbfleisch J. D., & Prentice R. L. (2002). The statistical analysis of failure time data (2nd ed.). John Wiley and Sons. 10.1002/9781118032985

[qlad082-B14] Kalbfleisch J. D., & Wolfe R. A. (2013). On monitoring outcomes of medical providers. Statistics in Biosciences, 5(2), 286–302. 10.1007/s12561-013-9093-x

[qlad082-B15] Kendzerska T., Zhu D. T., Gershon A. S., Edwards J. D., Peixoto C., Robillard R., & Kendall C. E. (2021). The effects of the health system response to the COVID-19 pandemic on chronic disease management: A narrative review. Risk Management and Healthcare Policy, 2021(14), 575–584. 10.2147/RMHP.S293471 PMC7894869 33623448

[qlad082-B16] Lawless J. F., & Nadeau C. (1995). Some simple robust methods for the analysis of recurrent events. Technometrics, 37(2), 158–168. 10.1080/00401706.1995.10484300

[qlad082-B17] Lin D. Y., Wei L. J., Yang I., & Ying Z. (2000). Semiparametric regression for the mean and rate functions of recurrent events. Journal of the Royal Statistical Society: Series B (Statistical Methodology), 62(4), 711–730. 10.1111/1467-9868.00259

[qlad082-B18] Liu D., Schaubel D. E., & Kalbfleisch J. D. (2012). Computationally efficient marginal models for clustered recurrent event data. Biometrics, 68(2), 637–647. 10.1111/j.1541-0420.2011.01676.x 21957989 PMC3384760

[qlad082-B19] Park S., & Hendry D. J. (2015). Reassessing Schoenfeld residual tests of proportional hazards in political science event history analyses. American Journal of Political Science, 59(4), 1072–1087. ISSN 1540–5907 10.1111/ajps.12176

[qlad082-B20] Prentice R. L., & Kalbfleisch J. D. (2003). Mixed discrete and continuous Cox regression model. Lifetime Data Analysis, 9(2), 195–210. 10.1023/A:1022935019768 12735496

[qlad082-B21] Roessler M., Schmitt J., Schoffer O., & Goudon T. (2021). Can we trust the standardized mortality ratio? A formal analysis and evaluation based on axiomatic requirements. PLOS One, 16(9), e0257003. 10.1371/journal.pone.0257003 34492062 PMC8423297

[qlad082-B22] Schoenfeld D. (1982). Partial residuals for the proportional hazards regression model. Biometrika, 69(1), 239–241. 10.1093/biomet/69.1.239

[qlad082-B23] The Council of State Governments . (2021). *COVID-19 resources for state leaders–executive orders*. https://web.csg.org/covid19/executive-orders/.

[qlad082-B24] United States Renal Data System. (2020). 2019 USRDS Annual Data Report: Epidemiology of kidney disease in the United States. *American Journal of Kidney Diseases*, *75*(1), A6–A7. 10.1053/j.ajkd.2019.09.003

[qlad082-B25] United States Renal Data System . (2021). *2020 USRDS Annual Data Report: Epidemiology of kidney disease in the United States*. National Institutes of Health, National Institute of Diabetes and Digestive and Kidney Diseases.

[qlad082-B26] Wu W., Taylor J. M. G., Brouwer A. F., Luo L., Kang J., Jiang H., & He K. (2022). Scalable proximal methods for cause-specific hazard modeling with time-varying coefficients. Lifetime Data Analysis, 28(2), 194–218. 10.1007/s10985-021-09544-2 35092553 PMC9201734

