# Supplementary material for: Models and methods for analysing clustered recurrent hospitalisations in the presence of COVID-19 effects
Source: J R Stat Soc Ser C Appl Stat. 2023 Sep 6;73(1):28–46. doi: 10.1093/jrsssc/qlad082 (PMC10782460; doi:10.1093/jrsssc/qlad082)
Supplement: qlad082_Supplementary_Data [file qlad082_supplementary_data.pdf]

Supplemental Materials

Xuemei Ding, Kevin He and Jack Kalbfleisch

Contents

1 Tables and Figures **2**

2 Over-dispersion method **13**

3 Mathematical details of computing the standard error of $\hat{\beta}$ **13**

4 Comments on the mortality model results **14**

1 Tables and Figures

Table S1: Accuracy of β estimation from the proposed algorithm in Scenario 3 (Independent) and 4 (Correlated). Results were from 1,000 repetitions. The model estimates of our proposed algorithm were almost the same as estimates from *survival* using *factor* argument and were similar to the estimates from *survival* using *strata* argument. The time and memory used in our proposed algorithm were much smaller to reach similar estimates. The robust estimator for the variance achieved better results than other methods when there was correlation. ESD: empirical standard deviation; MSE: mean square root error; ASE: the naive estimate of the asymptotic standard error; CP: coverage probability; sASE: sandwich estimator of the asymptotic standard error; sCP: coverage probability based on the sandwich estimator.

		Bias	ESD	MSE	ASE	CP	sASE	sCP
Independent	beta1	<0.001	0.043	0.002	0.042	0.941	0.042	0.937
	beta2	0.001	0.042	0.002	0.043	0.956	0.042	0.952
	beta3	0.001	0.042	0.002	0.043	0.953	0.042	0.948
	beta4	-0.003	0.044	0.002	0.043	0.934	0.042	0.928
	beta5	<0.001	0.042	0.002	0.043	0.943	0.042	0.939
	beta6	-0.001	0.031	0.001	0.031	0.954	0.030	0.949
	beta7	<0.001	0.032	0.001	0.032	0.950	0.032	0.947
	beta8	<0.001	0.026	0.001	0.026	0.948	0.025	0.940
	beta9	-0.001	0.026	0.001	0.026	0.944	0.025	0.941
	beta10	-0.002	0.026	0.001	0.026	0.947	0.026	0.940
Correlated	beta1	-0.003	0.067	0.005	0.043	0.780	0.069	0.957
	beta2	0.002	0.072	0.005	0.043	0.755	0.069	0.943
	beta3	<0.001	0.071	0.005	0.043	0.750	0.069	0.943
	beta4	-0.001	0.070	0.005	0.043	0.769	0.069	0.951
	beta5	-0.004	0.070	0.005	0.043	0.760	0.069	0.946
	beta6	<0.001	0.053	0.003	0.031	0.743	0.051	0.941
	beta7	0.001	0.050	0.002	0.032	0.785	0.048	0.943
	beta8	-0.002	0.043	0.002	0.026	0.753	0.042	0.941
	beta9	-0.004	0.043	0.002	0.026	0.748	0.041	0.941
	beta10	-0.003	0.041	0.002	0.026	0.795	0.041	0.952

Table S2: Continued from Table S1: Accuracy of β estimation from the *survival* package.

		survival using *factor*					*survival* using *strata*				
		Bias	ESD	MSE	ASE	CP	Bias	ESD	MSE	ASE	CP
Independent	beta1	<0.001	0.042	0.002	0.042	0.945	<0.001	0.043	0.002	0.042	0.941
	beta2	0.001	0.042	0.002	0.042	0.958	0.001	0.042	0.002	0.043	0.956
	beta3	<0.001	0.042	0.002	0.042	0.953	0.001	0.042	0.002	0.043	0.953
	beta4	-0.003	0.044	0.002	0.042	0.935	-0.003	0.044	0.002	0.043	0.934
	beta5	<0.001	0.042	0.002	0.042	0.945	<0.001	0.042	0.002	0.043	0.943
	beta6	-0.001	0.031	0.001	0.031	0.956	-0.001	0.031	0.001	0.031	0.954
	beta7	<0.001	0.032	0.001	0.032	0.951	<0.001	0.032	0.001	0.032	0.950
	beta8	<0.001	0.026	0.001	0.026	0.945	<0.001	0.026	0.001	0.026	0.948
	beta9	-0.001	0.026	0.001	0.025	0.947	-0.001	0.026	0.001	0.026	0.944
	beta10	-0.002	0.026	0.001	0.026	0.950	-0.002	0.026	0.001	0.026	0.947
Correlated	beta1	-0.003	0.067	0.005	0.042	0.780	-0.003	0.067	0.005	0.043	0.780
	beta2	0.002	0.072	0.005	0.043	0.749	0.002	0.072	0.005	0.043	0.755
	beta3	<0.001	0.071	0.005	0.043	0.756	<0.001	0.071	0.005	0.043	0.750
	beta4	-0.001	0.070	0.005	0.042	0.766	-0.001	0.070	0.005	0.043	0.769
	beta5	-0.005	0.070	0.005	0.042	0.761	-0.004	0.070	0.005	0.043	0.760
	beta6	<0.001	0.053	0.003	0.031	0.743	<0.001	0.053	0.003	0.031	0.743
	beta7	0.001	0.050	0.002	0.032	0.778	0.001	0.050	0.002	0.032	0.785
	beta8	-0.002	0.043	0.002	0.026	0.759	-0.002	0.043	0.002	0.026	0.753
	beta9	-0.003	0.043	0.002	0.025	0.747	-0.004	0.043	0.002	0.026	0.748
	beta10	-0.003	0.041	0.002	0.026	0.793	-0.003	0.041	0.002	0.026	0.795

Table S3: Results of α_F estimation from the *survival* package using *factor* argument in Scenario 5 (Independent) and 6 (Correlated). Results were from 1,000 repetitions. ESD: empirical standard deviation; ASE: the naive estimate of the asymptotic standard error; CP: coverage probability; sASE: sandwich estimator of the asymptotic standard error; sCP: coverage probability based on the sandwich estimator. pASE: asymptotic standard error adjusted by over-dispersion factor based on Pearson residual; pCP: coverage probability based on estimated over-dispersion variance.

	Size	Bias	ESD	ASE	CP
	50	0.005	0.212	0.209	0.956
	100	0.017	0.195	0.190	0.950
	200	0.013	0.186	0.181	0.953
	300	0.014	0.179	0.178	0.954
Independent	500	0.024	0.176	0.176	0.960
	1000	0.015	0.173	0.172	0.949
	2000	0.019	0.178	0.171	0.948
	3000	0.011	0.174	0.170	0.944
	5000	0.021	0.175	0.171	0.949
	50	0.011	0.328	0.213	0.806
	100	0.019	0.273	0.193	0.842
	200	0.002	0.272	0.181	0.821
	300	0.018	0.258	0.179	0.830
Correlated	500	0.032	0.262	0.177	0.813
	1000	0.026	0.257	0.174	0.836
	2000	0.031	0.254	0.174	0.828
	3000	0.027	0.239	0.172	0.847
	5000	0.030	0.251	0.172	0.824

Table S4: Computation time results of simulation data for a list of different numbers of threads. Serial is the code without parallel computing. Speedup is the time used by serial code divided by the time used by a given number of threads. Efficiency is the speedup divided by the number of threads.

Number of threads	Time (s)	Speedup	Efficiency
Serial	7590.3	-	-
2	4261.8	1.78099	0.890494
4	2214.4	3.42768	0.856920
8	1102.6	6.88369	0.860461
16	575.3	13.19334	0.824584

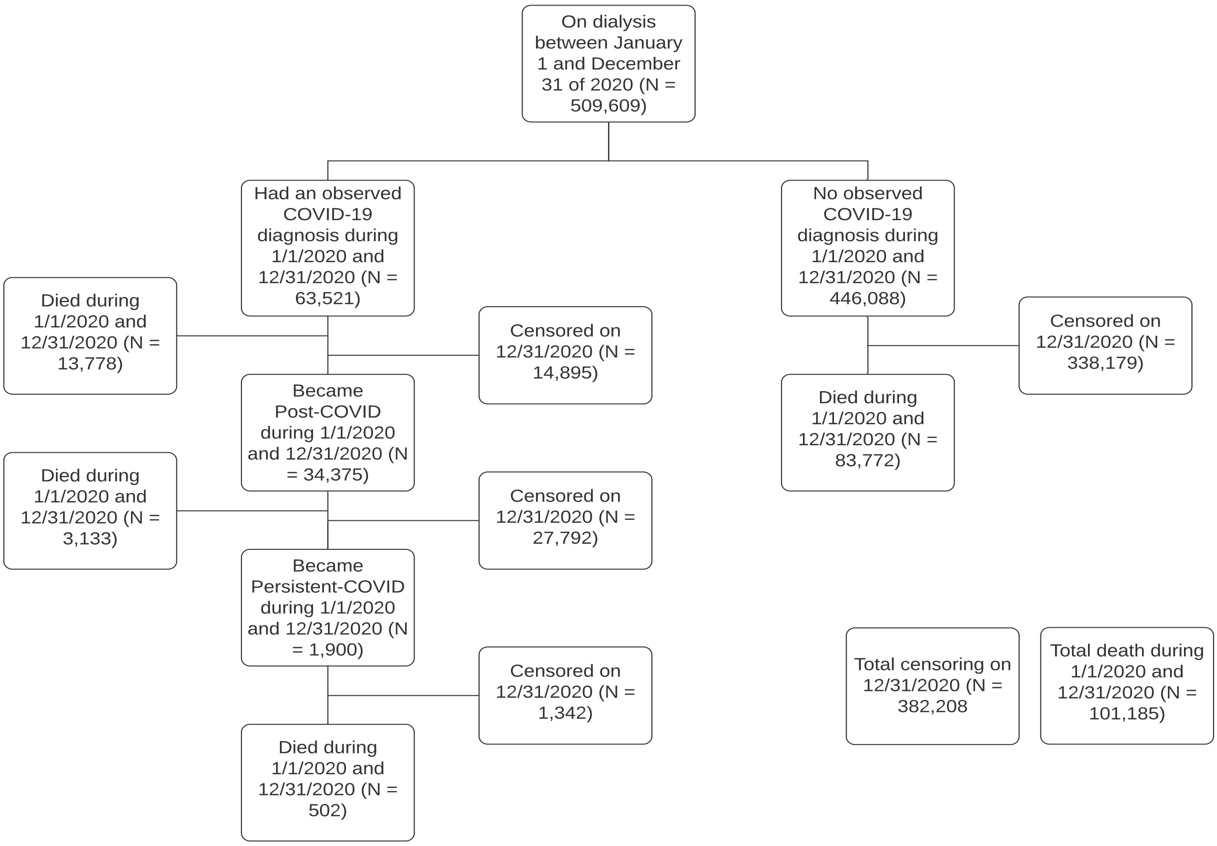

Figure S1: A flow chart of the number of patients at risk, in each COVID-19 stage, and who died.

Table S5: Characteristics of patients by COVID-19 stages.

	No-COVID, N = 446,088	COVID, N = 63,521	P-value*	Post-COVID, N = 34,375	Persistent-COVID, N = 1,900	Death, N = 101,185
Age in 2020: mean (sd)	64.7 (14)	64.6 (13.5)	0.6006	62.9 (13.7)	63.6 (13.6)	69.8 (12.3)
Female	42.80%	44.70%	<0.0001	45.90%	44.60%	42.90%
Race						
White	59.90%	57.30%	<0.0001	54%	58.90%	66.10%
Black	32.70%	36.10%	<0.0001	39.90%	34.90%	27.80%
Asian/Pacific Islander	5.90%	4.50%	<0.0001	4%	4.10%	4.60%
Others	1.40%	2.20%	<0.0001	2.10%	2.10%	1.50%
Ethnicity						
Non-Hispanic	82.80%	77%	<0.0001	76.60%	72.10%	84.30%
Hispanic	16.60%	22.40%	<0.0001	22.80%	27.50%	15.20%
Unknown	0.60%	0.60%	0.3819	0.60%	0.50%	0.50%
Cause of ESRD: diabetes	46.40%	53.30%	<0.0001	51.20%	55.70%	51.60%
Missing: Primary disease causing ESRD	0.90%	1.10%	<0.0001	1.30%	1.30%	1%
Proportion of days with Medicare Advantages coverage: mean (sd)	0.3 (0.4)	0.2 (0.4)	<0.0001	0.2 (0.4)	0.2 (0.4)	0.3 (0.4)
Time since ESRD						
<90 days	14.40%	12.80%	0.0837	14.10%	13.50%	13.10%
90 days - 1 year	12.30%	11.10%	0.0834	10.90%	11.00%	11.70%
1 year – 3 years	25.70%	25.40%	0.1063	25.00%	25.60%	24.80%
>3 years	47.60%	50.70%	0.1232	50.00%	49.80%	50.50%
BMI						
$\leq$ 18.4	2.80%	2.30%	<0.0001	2.40%	2.60%	3%
18.5-24.9	25.20%	23.10%	<0.0001	23.70%	25.20%	26.60%
25-29.9	27.30%	26.60%	2.00E-04	26.10%	27.30%	27.50%
$\geq$ 30	44.70%	47.90%	<0.0001	47.80%	44.90%	42.80%
Incident Comorbidity Atherosclerotic heart disease	11.50%	11.80%	0.0436	10.80%	10.90%	15.50%
Other cardiac disease	16.10%	16.20%	0.5285	15.30%	15.60%	20.90%
Congestive heart failure	23.90%	26.60%	<0.0001	25.60%	28.10%	31.30%
Inability to ambulate	4%	6.50%	<0.0001	6.60%	7.20%	7%
Chronic obstructive pulmonary disease	6.70%	7%	0.0011	6.40%	7.40%	9.90%
Inability to transfer	1.90%	3.40%	<0.0001	3.50%	4.40%	3.60%
Malignant neoplasm, Cancer	5.60%	4.50%	<0.0001	4.10%	4.10%	7.20%
Diabetes	13.80%	14.80%	<0.0001	14.90%	14.70%	15.20%
Peripheral vascular disease	7.90%	8.80%	<0.0001	8.50%	10%	10.80%

Cerebrovascular disease, CVA, TIA	7.40%	9.20%	<0.0001	9.20%	10.30%	9.40%
Current smoker	6.10%	4.70%	<0.0001	4.80%	5.60%	6.40%
Alcohol	1.20%	1.10%	0.2939	1.20%	1.50%	1.30%
Drug dependence	1.10%	1.10%	0.835	1.20%	1.30%	1%
At least one incident comorbidity available	75.30%	79.60%	<0.0001	77.90%	82.20%	83.40%
Missing 2728 Form	1.20%	1.30%	0.0273	1.50%	1.50%	1.20%
Prevalent Comorbidity						
Diabetes without complications	8.80%	12.50%	<0.0001	11.90%	17.50%	15%
Diabetes with complications	29.40%	38.10%	<0.0001	36.60%	47.60%	44.70%
Glucocorticoid deficiency	0.50%	0.60%	0.036	0.60%	1.30%	1.10%
Malnutrition / Cachexia	4.10%	4.80%	<0.0001	4.70%	6.90%	9.20%
Other specified disorders of metabolism	0.90%	1.20%	<0.0001	1.20%	1.50%	1.40%
Pancytopenia	1.70%	2%	<0.0001	2%	3.30%	3.30%
Primary hypercoagulable	0.50%	0.60%	0.0029	0.70%	0.90%	0.70%
Dementia	2.90%	5.40%	<0.0001	5%	6.70%	7.60%
Opioid Dependence	0.70%	0.90%	<0.0001	1%	1.80%	1.30%
Epilepsy	3.10%	4.70%	<0.0001	4.80%	6.30%	5.40%
Bipolar Disorder	0.80%	1.40%	<0.0001	1.50%	2.10%	1.30%
Major depressive affective disorder	8.10%	11.30%	<0.0001	11.10%	15%	13.70%
Coma	0.60%	0.80%	<0.0001	0.80%	0.90%	1.40%
hypertensive heart disease with heart failure	0.80%	1%	<0.0001	1%	1.90%	1.50%
Myocardial Infarction	5.10%	5.90%	<0.0001	5.50%	8.50%	9.90%
Coronary Atherosclerosis	5%	6.10%	<0.0001	5.90%	8.80%	9%
pulmonary embolism and infarction	0.50%	0.60%	5.00E-04	0.60%	0.90%	0.90%
Cardiomyopathy	5.10%	5.90%	<0.0001	5.80%	8.30%	9.20%
Paroxysmal Tachycardia	1.80%	2%	0.0317	1.90%	3.50%	3.90%
Atrial fibrillation	11.90%	13.10%	<0.0001	11.90%	15.70%	22.60%
Sinoatrial node dysfunction	3.10%	3.70%	<0.0001	3.40%	4.30%	5.60%
Acute Cerebrovascular Disease	1.50%	1.90%	<0.0001	1.90%	2.40%	2.90%
Peripheral and Visceral Atherosclerosis	5.40%	6.20%	<0.0001	5.90%	9%	10.30%

Venous Thromboembolism	1.80%	2.30%	<0.0001	2.40%	3.90%	3.20%
Chronic Obstructive Pulmonary Disease	10.10%	12.50%	<0.0001	11.60%	16.30%	19.10%
Asthma	9%	11.30%	<0.0001	10.60%	14.80%	17.30%
Respiratory Failure	12.60%	15.30%	<0.0001	14.50%	20.80%	23.20%
Ileus and Intestinal Obstruction	1.30%	1.70%	<0.0001	1.70%	1.90%	2.30%
Cirrhosis of Liver	2%	2.30%	<0.0001	2.20%	4.20%	4.20%
Other Liver Disease	1%	1.10%	0.0022	1.10%	2.20%	2.30%
Pancreatitis	0.50%	0.60%	<0.0001	0.70%	1.30%	0.80%
Chronic Skin Ulcer	5.50%	7.90%	<0.0001	7.40%	11.30%	12.10%
Systemic lupus erythematosus and connective tissue disorders	1%	0.90%	0.0769	1%	1.60%	1%
Rheumatoid Arthritis	0.90%	1%	0.0017	1%	1.50%	1.40%
Gangrene	2.50%	3.70%	<0.0001	3.60%	5.50%	5.40%
Solid Organ Transplant	0.60%	0.60%	0.6928	0.50%	0.60%	0.80%
Ileostomy / Colostomy	0.60%	0.80%	<0.0001	0.80%	1.30%	1.10%
Toe(s) amputation	1.60%	2.40%	<0.0001	2.40%	3.50%	2.80%
Above knee amputation	0.80%	1.30%	<0.0001	1.30%	2.10%	1.80%
Long-term use of insulin	16%	22.20%	<0.0001	21.30%	29.60%	25.20%
Less than 6 months of Medicare covered months in the prior calendar year	17.40%	16.70%	<0.0001	18.10%	14.90%	12.60%

Table S6: Model fitting results, continued from Table 3 in the main paper

	Hospitalization			Mortality		
Parameter	Model Estimate	p-value	RR and 95% CI	Model Estimate	p-value	HR and 95% CI
Nursing Home care (reference: 0 Nursing Home days*)	-	-	-	-	-	-
Short-term Nursing Home care:0 < total Nursing Home days < 90	0.16	<0.001	$_{1.09}1.17_{1.26}$	0.42	<0.001	$_{1.49}1.52_{1.55}$
Long-term Nursing Home care: total Nursing Home days $\geq$ 90	-0.01	0.417	$_{0.93}0.99_{1.06}$	0.36	<0.001	$_{1.40}1.43_{1.47}$
Incident Comorbidity	-	-	-	-	-	-

Atherosclerotic heart disease	0.04	0.151	$_{0.97}1.04_{1.11}$	0.07	<0.001	$_{1.05}1.07_{1.09}$
Other cardiac disease	0.03	0.214	$_{0.96}1.03_{1.10}$	0.07	<0.001	$_{1.06}1.08_{1.09}$
Congestive heart failure	0.03	0.182	$_{0.96}1.03_{1.11}$	0.11	<0.001	$_{1.10}1.12_{1.13}$
Inability to ambulate	0.03	0.172	$_{0.96}1.03_{1.11}$	0.12	<0.001	$_{1.09}1.12_{1.16}$
Chronic obstructive pulmonary disease	0.06	0.051	$_{0.99}1.06_{1.14}$	0.07	<0.001	$_{1.05}1.07_{1.10}$
Inability to transfer	0.06	0.034	$_{1.00}1.07_{1.14}$	0.09	<0.001	$_{1.05}1.10_{1.15}$
Malignant neoplasm, Cancer	0.06	0.054	$_{0.99}1.06_{1.13}$	0.13	<0.001	$_{1.11}1.14_{1.17}$
Diabetes	-0.02	0.241	$_{0.91}0.98_{1.05}$	0.04	<0.001	$_{1.02}1.05_{1.07}$
Peripheral vascular disease	0.05	0.1	$_{0.98}1.05_{1.12}$	0.08	<0.001	$_{1.06}1.08_{1.11}$
Cerebrovascular disease, CVA, TIA	0.02	0.272	$_{0.95}1.02_{1.09}$	0.00	0.437	$_{0.98}1.00_{1.02}$
Current smoker	0.10	0.002	$_{1.03}1.11_{1.19}$	0.15	<0.001	$_{1.13}1.16_{1.19}$
Alcohol	0.07	0.021	$_{1.00}1.07_{1.15}$	0.02	0.259	$_{0.96}1.02_{1.08}$
Drug dependence	0.22	<0.001	$_{1.16}1.25_{1.34}$	0.08	0.012	$_{1.01}1.08_{1.16}$
At least one incident comorbidity available	0.07	0.024	$_{1.00}1.07_{1.15}$	0.11	<0.001	$_{1.09}1.12_{1.14}$
Missing 2728 Form	0.00	0.495	$_{0.93}1.00_{1.07}$	0.32	<0.001	$_{1.22}1.38_{1.57}$
Less than 6 months of Medicare covered months in the prior calendar year	0.43	<0.001	$_{1.44}1.54_{1.65}$	0.35	<0.001	$_{1.38}1.41_{1.45}$
Prevalent Comorbidity	-	-	-	-	-	-
Diabetes without complications	0.07	0.029	$_{1.00}1.07_{1.15}$	0.03	0.004	$_{1.01}1.03_{1.05}$
Diabetes with complications	0.22	<0.001	$_{1.17}1.25_{1.34}$	0.08	<0.001	$_{1.06}1.08_{1.11}$
Glucocorticoid deficiency	0.18	<0.001	$_{1.12}1.20_{1.28}$	0.34	<0.001	$_{1.32}1.41_{1.50}$
Malnutrition / Cachexia	0.11	0.001	$_{1.05}1.12_{1.20}$	0.30	<0.001	$_{1.31}1.35_{1.38}$
Other specified disorders of metabolism	0.19	<0.001	$_{1.13}1.21_{1.29}$	0.02	0.235	$_{0.96}1.02_{1.08}$
Pancytopenia	0.19	<0.001	$_{1.13}1.21_{1.29}$	0.19	<0.001	$_{1.16}1.21_{1.26}$
Primary hypercoagulable state	0.11	0.001	$_{1.04}1.11_{1.19}$	0.02	0.305	$_{0.94}1.02_{1.10}$
Dementia	0.04	0.12	$_{0.97}1.04_{1.12}$	0.15	<0.001	$_{1.14}1.17_{1.20}$
Opioid Dependence	0.36	<0.001	$_{1.34}1.44_{1.54}$	0.16	<0.001	$_{1.11}1.18_{1.25}$
Epilepsy	0.19	<0.001	$_{1.13}1.21_{1.30}$	0.10	<0.001	$_{1.07}1.11_{1.14}$
Bipolar Disorder	0.26	<0.001	$_{1.21}1.30_{1.39}$	0.01	0.435	$_{0.95}1.01_{1.07}$
Major depressive affective disorder	0.23	<0.001	$_{1.17}1.25_{1.34}$	0.06	<0.001	$_{1.04}1.06_{1.08}$
Coma	-0.06	0.059	$_{0.88}0.95_{1.01}$	0.25	<0.001	$_{1.20}1.28_{1.36}$
hypertensive heart disease with heart failure	0.12	<0.001	$_{1.06}1.13_{1.21}$	0.02	0.26	$_{0.96}1.02_{1.08}$
Myocardial Infarction	0.15	<0.001	$_{1.09}1.16_{1.25}$	0.21	<0.001	$_{1.21}1.24_{1.27}$
Coronary Atherosclerosis	0.17	<0.001	$_{1.11}1.19_{1.27}$	0.09	<0.001	$_{1.06}1.09_{1.12}$
pulmonary embolism and infarction	0.10	0.002	$_{1.03}1.11_{1.19}$	0.10	0.003	$_{1.03}1.11_{1.19}$
Pulmonary Heart Disease	0.12	0.001	$_{1.05}1.12_{1.20}$	0.16	<0.001	$_{1.15}1.17_{1.20}$

Cardiomyopathy	0.13	<0.001	$_{1.06}1.13_{1.22}$	0.15	<0.001	$_{1.13}1.16_{1.19}$
Paroxysmal Tachycardia	0.08	0.012	$_{1.01}1.08_{1.16}$	0.19	<0.001	$_{1.16}1.21_{1.25}$
Atrial fibrillation	0.10	0.002	$_{1.03}1.11_{1.19}$	0.18	<0.001	$_{1.18}1.20_{1.22}$
Sinoatrial node dysfunction	0.03	0.232	$_{0.96}1.03_{1.10}$	-0.03	0.047	$_{0.95}0.98_{1.00}$
Acute Cerebrovascular Disease	0.04	0.104	$_{0.98}1.05_{1.12}$	0.13	<0.001	$_{1.09}1.14_{1.18}$
Peripheral and Visceral Atherosclerosis	0.14	<0.001	$_{1.08}1.16_{1.24}$	0.13	<0.001	$_{1.11}1.14_{1.17}$
Venous Thromboembolism	0.15	<0.001	$_{1.09}1.17_{1.25}$	0.12	<0.001	$_{1.08}1.13_{1.17}$
Chronic Obstructive Pulmonary Disease	0.13	<0.001	$_{1.07}1.14_{1.22}$	0.15	<0.001	$_{1.10}1.16_{1.22}$
Asthma	0.07	0.022	$_{1.00}1.07_{1.15}$	-0.02	0.209	$_{0.93}0.98_{1.03}$
Respiratory Failure	0.16	<0.001	$_{1.10}1.18_{1.26}$	0.18	<0.001	$_{1.17}1.20_{1.22}$
Ileus and Intestinal Obstruction	0.06	0.04	$_{0.99}1.06_{1.14}$	-0.05	0.018	$_{0.91}0.95_{1.00}$
Cirrhosis of Liver	0.21	<0.001	$_{1.15}1.23_{1.32}$	0.32	<0.001	$_{1.32}1.38_{1.44}$
Other Liver Disease	0.23	<0.001	$_{1.17}1.26_{1.35}$	0.28	<0.001	$_{1.25}1.32_{1.39}$
Pancreatitis	0.36	<0.001	$_{1.34}1.44_{1.54}$	0.10	0.005	$_{1.02}1.10_{1.18}$
Chronic Skin Ulcer	0.09	0.005	$_{1.02}1.10_{1.17}$	0.22	<0.001	$_{1.21}1.25_{1.28}$
Systemic lupus erythematosus and connective tissue disorders	0.21	<0.001	$_{1.15}1.23_{1.32}$	0.25	<0.001	$_{1.20}1.28_{1.37}$
Rheumatoid Arthritis	0.12	0.001	$_{1.05}1.12_{1.20}$	0.06	0.012	$_{1.01}1.06_{1.12}$
Gangrene	0.03	0.223	$_{0.96}1.03_{1.10}$	0.15	<0.001	$_{1.12}1.16_{1.20}$
Solid Organ Transplant	0.12	0.001	$_{1.05}1.12_{1.20}$	-0.03	0.256	$_{0.90}0.98_{1.05}$
Ileostomy / Colostomy	0.18	<0.001	$_{1.11}1.19_{1.28}$	-0.03	0.162	$_{0.91}0.97_{1.03}$
Toe(s) amputation	0.08	0.011	$_{1.01}1.08_{1.16}$	0.01	0.39	$_{0.96}1.01_{1.05}$
Above knee amputation	0.04	0.14	$_{0.97}1.04_{1.11}$	0.17	<0.001	$_{1.13}1.19_{1.25}$
Long-term use of insulin	0.09	0.008	$_{1.02}1.09_{1.17}$	0.02	0.023	$_{1.00}1.02_{1.04}$

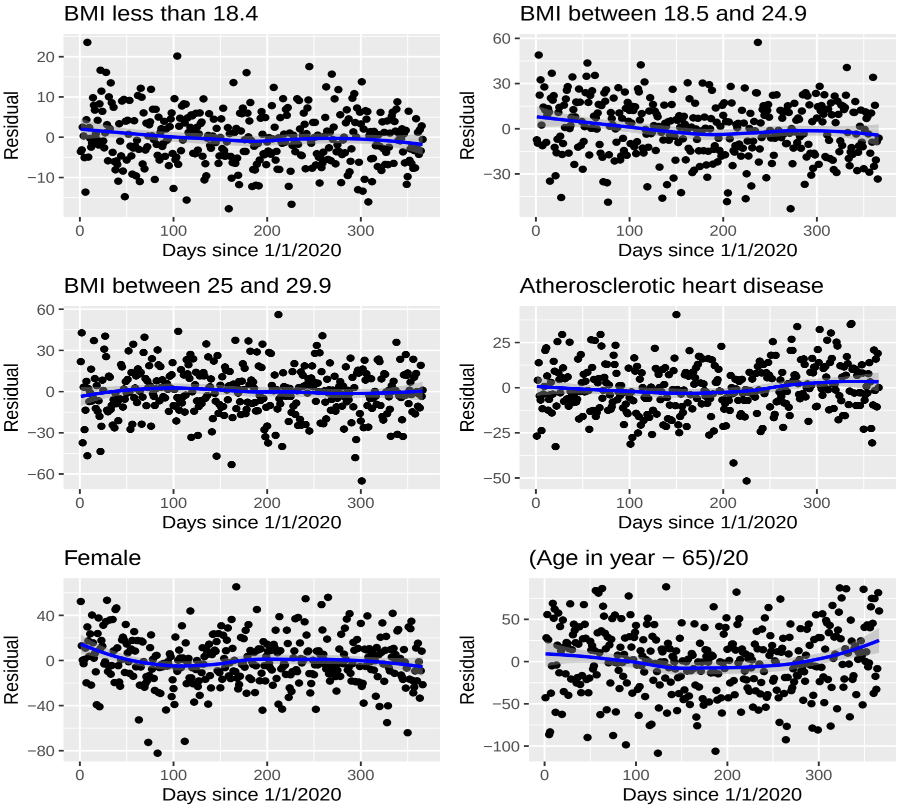

Figure S2: Example Schoenfeld residual plots. The dots are the Schoenfeld residuals for tied data of each calendar day. Taking age as an example, for every time point, we sum the age of all patients who have events at that time point, and minus the number of events times the average age of all at-risk patients at that time point. The curve is the smoothed line, and the gray area is the normal-determined 95% confidence interval.

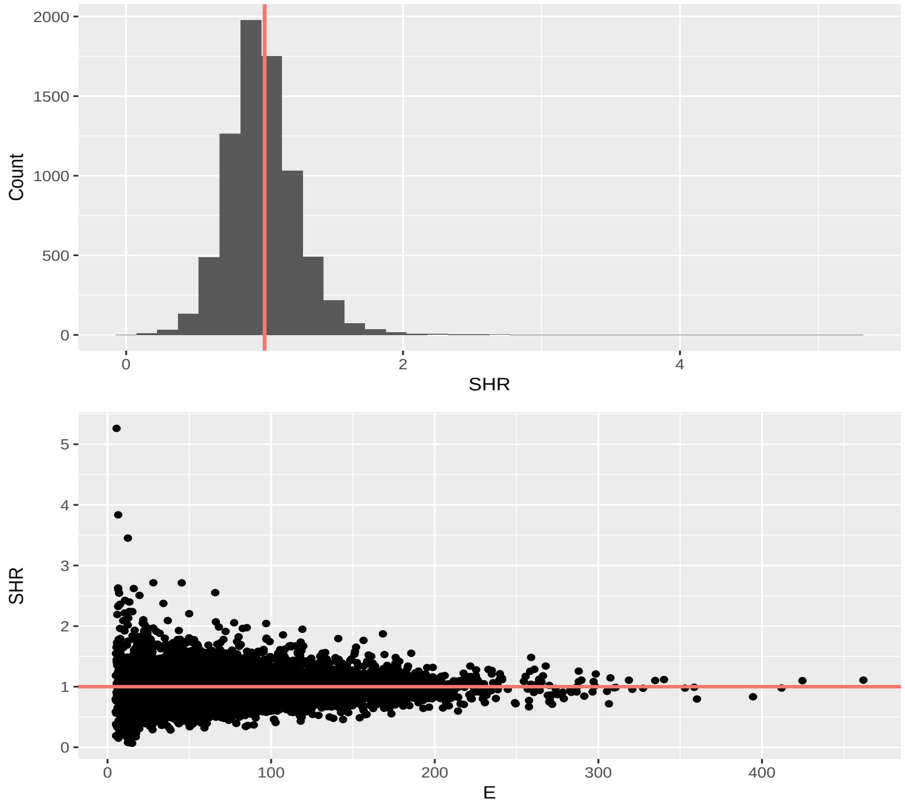

Figure S3: Histogram plot (above) and funnel plot (bottom) for the standardized hospitalization ratio (SHR). The pink line indicates SHR = 1. The facilities with expected number of hospitalizations under the null (E) greater than 800 or smaller than 5, or the observed number of hospitalizations (O) equal to 0 are excluded in the plots.

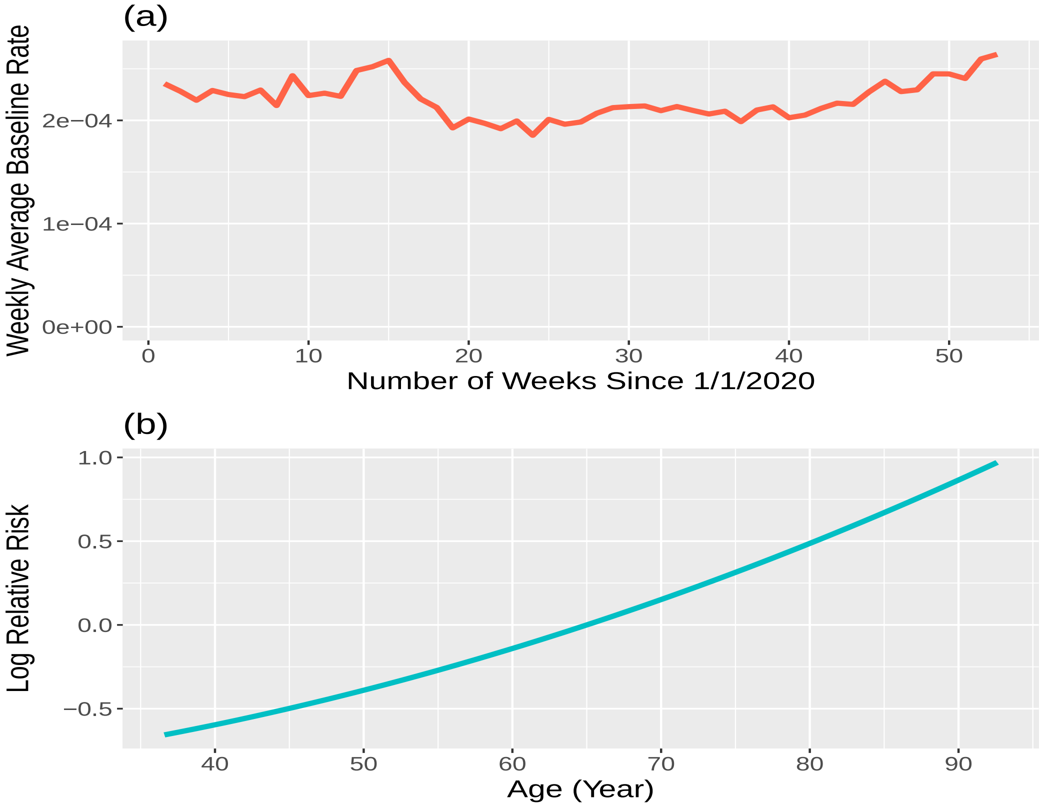

Figure S4: a. The estimated baseline death rate function averaged by week. b. The estimated age effect curve.

2 Over-dispersion method

Another way to improve the naive estimator is to allow over-dispersion, i.e., let $V_j = \phi \frac{1}{O_j}$, where ϕ is the over-dispersion scaling factor. We used the Pearson residual to calculate the scaling factor ϕ:

$$\phi = \frac{\sum_j \sum_{G_i=j} [(O_{ij} - E_{ij}\frac{O_j}{E_j})^2 / E_{ij}\frac{O_j}{E_j}]}{n - F}.$$

3 Mathematical details of computing the standard error of $\hat{\beta}$

The matrix of negative partial derivatives of $U(\boldsymbol{\beta})$ is

$$\mathcal{I}(\boldsymbol{\beta}) = \sum_{j=1}^{F} \sum_{m=1}^{M} dN^j(t_m) \frac{S_j^{(2)}(t_m) S_j^{(0)}(t_m) - S_j^{(1)}(t_m)^{\otimes 2}}{S_j^{(0)}(t_m)^2}. \tag{S1}$$

4 Comments on the mortality model results

On one hand, similar to the hospitalization model, in the no-COVID group, Black and Asian/Pacific Islander had lower risks (0.72 and 0.73, respectively, $p < 0.001$) compared to White patients, and Hispanics had a lower hazard ratio (0.74, $p < 0.001$) compared to non-Hispanics. On the other hand, the COVID1 effect's estimate in the mortality model was much smaller than that in the hospitalization model, while the COVID2 effect's estimate was much higher; in the no-COVID group, females had a lower hazard ratio of mortality (0.93, $p < 0.001$) compared to males, but a higher relative rate of hospitalization.
